# Genetic Insights into Brain Morphology: a Genome-Wide Association Study of Cortical Thickness and T_1_-Weighted MRI Gray Matter-White Matter Intensity Contrast

**DOI:** 10.1007/s12021-025-09722-9

**Published:** 2025-04-01

**Authors:** Nicholas J. Kim, Nahian F. Chowdhury, Kenneth H. Buetow, Paul M. Thompson, Andrei Irimia

**Affiliations:** 1 University of Southern California (Alfred E. Mann Department of Biomedical Engineering, Viterbi School of Engineering), Los Angeles, CA USA; 2 University of Southern California (Ethel Percy Andrus Gerontology Center, Leonard Davis School of Gerontology), Los Angeles, CA USA; 3 Arizona State University (School of Life Sciences Center for Social Dynamics and Complexity), Tempe, AZ USA; 4 University of Southern California (Mark and Mary Stevens Neuroimaging and Informatics Institute), Marina del Rey, Los Angeles, CA USA; 5 University of Southern California (Department of Quantitative & Computational Biology, Dornsife College of Arts and Sciences), Los Angeles, CA USA; 6 King’s College London (Centre for Healthy Brain Aging, Institute of Psychiatry, Psychology & Neuroscience), London, England UK

**Keywords:** T1-weighted MRI, Cortical thickness, Gray-white matter contrast, Brain morphology, Genome-wide association study, Dimensionality reduction, UMAP

## Abstract

In *T*_*1*_-weighted magnetic resonance imaging (MRI), cortical thickness (CT) and gray-white matter contrast (GWC) capture brain morphological traits and vary with age-related disease. To gain insight into genetic factors underlying brain structure and dynamics observed during neurodegeneration, this genome-wide association study (GWAS) quantifies the relationship between single nucleotide polymorphisms (SNPs) and both CT and GWC in UK Biobank participants (*N* = 43,002). To our knowledge, this is the first GWAS to investigate the genetic determinants of cortical *T*_*1*_-MRI GWC in humans. We found 251 SNPs associated with CT or GWC for at least 1% of cortical locations, including 42 for both CT and GWC; 127 for only CT; and 82 for only GWC. Identified SNPs include rs1080066 (THSB1, featuring the strongest association with both CT and GWC), rs13107325 (SLC39A8, linked to CT at the largest number of cortical locations), and rs864736 (KCNK2, associated with GWC at the largest number of cortical locations). Dimensionality reduction reveals three major gene ontologies constraining CT (neural signaling, ion transport, cell migration) and four constraining GWC (neural cell development, cellular homeostasis, tissue repair, ion transport). Our findings provide insight into genetic determinants of GWC and CT, highlighting pathways associated with brain anatomy and dynamics of neurodegeneration. These insights can assist the development of gene therapies and treatments targeting brain diseases.

**Supplementary Information:**

The online version contains supplementary material available at 10.1007/s12021-025-09722-9.

## Introduction

The morphology of the human brain is complex due to its intricate folding patterns (Prodromidou & Matsas, 2021), diverse cellular architecture (Prodromidou & Matsas, 2021), and regional specializations (Miterko et al., 2018). Because many such morphological brain traits are heritable, identifying the genetic variants (single-nucleotide polymorphisms, SNPs) constraining them can enhance our understanding of brain structure and the structural changes associated with age-related disease. In *T*_*1*_*-*weighted magnetic resonance imaging (MRI), two key anatomic variables whose dynamics capture genetic influences on brain morphology are cortical thickness (CT) and the gray (GM) / white matter (WM) intensity contrast (GWC).

The increasing availability of genotyped samples paired with MRIs, as in the UK Biobank (UKBB), enables genome-wide association studies (GWASs) to identify genetic variants linked to structural brain traits. Whereas previous GWASs identified many genetic determinants of CT, the genetic basis of GWC remains less explored (Panizzon et al., 2012). CT, the distance between pial and white matter surfaces (Fischl & Dale, 2000), has been linked to age-related cortical atrophy (Petersen et al., 2022) and neurodegeneration (Singh et al., 2006; Yau et al., 2018). GWC, defined as the normalized difference between GM and WM intensities, has also been implicated in aging (Davatzikos & Resnick, 2002) and neurodegeneration (Uribe et al., 2018). Although highly heritable, CT and GWC are constrained by distinct genetic mechanisms (Panizzon et al., 2012). In this study, we conduct a GWAS of CT and GWC across the cortical mantle in up to 43,002 UKBB individuals. Our analysis identifies 251 variants associated with CT or GWC in at least 1% of the cortical locations examined: 42 variants for both CT and GWC; 127 variants for only CT; and 82 variants for only GWC. Dimensionality reduction of CT and GWC reveals four prominent spatial patterns of genetic association shared by SNPs for each phenotype.

## Methods

### Participants

This research has been conducted using data from UKBB, a major biomedical database (http://www.ukbiobank.ac.uk), under application number 47656. The principles outlined in the Declaration of Helsinki and stipulated in the US Code of Federal Regulations (45 C.F.R. 46) were followed. Ethical approval for UKBB initiatives was obtained from the North West Multi-Centre Research Ethics Committee of the United Kingdom. All participants provided informed written consent. The study cohort comprises 43,030 cognitively normal individuals (22,679 females) aged 45 to 83 years (mean: 64.4 years (y); standard deviation: 7.7 y). The sex ratio is 1.11 females for every male. 28 participants were excluded due to missing scan data. Details on the UKBB infrastructure and recruitment are available elsewhere (Alfaro-Almagro et al., 2018).

### MRI Acquisition and Preprocessing

*T*_*1*_*-*weighted MRIs were acquired using a standard Siemens Skyra 3.0 T scanner with a voxel size 1 mm × 1 mm × 1 mm and a matrix size of 208 × 256 × 256 voxels. MRIs were preprocessed using FreeSurfer version 7.3.2 (FS) reconstructions generated by a UKBB workflow (Alfaro-Almagro et al., 2018). This process encompassed skull-stripping, motion correction, Talairach space transformation, and elimination of non-brain tissues. Nonuniform signal intensities were normalized using FS MRI-normalize, which scaled intensities for all voxels so that the mean WM intensity was a common value (Sled et al., 1998).

At 5,124 cortical locations, CT was computed as the distance between the GM-WM interface and the pial surface (Fischl & Dale, 2000). At each location, GWC was calculated by averaging *T*_*1*_*-*weighted MRI intensities of the GM across voxels between the GM-WM interface and the pial surface. This process utilized the FS GM mask to identify intensity values associated with GM voxels. Mesh representations of the GM/WM interface and pial surface were employed to calculate intensity values for each cortical location on the GM/WM surface. Correspondences between pial mesh vertices and WM mesh vertices were identified by computing the shortest distance between each WM vertex and the pial surface. For each pair of vertices across the two surfaces, GM intensities were extracted from all voxels found within a cylinder with bases centered on the vertex pair and with radius equal to the distance between WM and pial vertices. These intensities were averaged and assigned to the corresponding location on the GM/WM surface. GWC, calculated as $\frac{(WM-GM)}{(WM+GM)}$, was computed at each location on the GM/WM surface using the respective mean GM intensity and the mean WM intensity value used for FS scaling. To accommodate variations in brain morphology, the FS MRI-surf2surf function was used to project CT and GWC values onto the FS average cortical atlas (Fischl, 2012).

### Genetic Data

A total of 784,036 SNPs were examined across the genome. We used genotype data from UKBB SNPs imputed to the Haplotype Reference Consortium (UKBB Category 100319). UKBB quality control (QC), phasing, and imputation procedures are outlined in (Bycroft et al., 2018). We excluded (A) 18,876 SNPs for which data were available in fewer than 35,000 participants, (B) 95,267 SNPs for which over 99% of participants were monomorphic, (C) 4,873 SNPs with missing reference identifiers, and (D) 2,049 SNPs with erroneous reference allele information. Analyses involved the remaining 662,971 SNPs. Participants’ genotypes were coded according to their number of reference alleles (i.e., 0, 1, or 2 alleles with a reference nucleic acid) possessed by each participant at each SNP. SNPs were mapped to genes (both protein-coding and noncoding) using (A) prior research compiled by the National Human Genome Research Institute-European Bioinformatics Institute (NHGRI-EBI) GWAS catalog (Sollis et al., 2022) and (B) Bedtools’ closest function with the Genome Reference Consortium Human Build 37 (GRCh37) (Church et al., 2011; Quinlan & Hall, 2010). For the latter method, gene boundaries were defined from GRCh37 and SNPs were assigned to the overlapping gene with the closest boundary.

### GWAS

Linear regression assessed the dose–response relationship between (A) the number of reference alleles at a given SNP and (B) local CT. Linear regression also investigated the relationship between (A) the number of reference alleles at a given SNP and (B) local GWC. Covariates included age at MRI scan (from UKBB fields 34, 52 and 53), sex (field 31), and the first five genetic principal components to control for population structure (field 22009) (Tucker et al., 2014). Imaging center (UKBB field 54) was excluded to improve computational efficiency, as the UK Biobank has implemented extensive quality control procedures to harmonize data across sites (Alfaro-Almagro et al., 2018). Additionally, prior research using UKBB data has found minimal site-related variability in GWAS (Dor et al., 2023). An analysis of variance quantifying the impact of imaging center on phenotypic variability showed that scanner site accounted for an average of only 0.17% of the variance in CT across 5,124 locations (range: 0.00%–1.57%, SD: 0.11%) and 0.20% of the variance in GWC (range: 0.02%–2.56%, SD: 0.18%) (Supplementary Figs. 1 and 2). Because not all participants possessed genotype data for all 662,971 SNPs, sample sizes were adjusted for each SNP to exclude individuals with missing SNP data. Thus, sample sizes used for GWAS ranged from 35,000 to 41,798 participants (mean sample size: 41,419; standard deviation: 874). With a null hypothesis of no linear correlation, *p*-values were calculated using a two-tailed *t*-test comparing *β* regression coefficients. Genome-wide statistical significance was set at *p* < 0.05 with Benjamini–Hochberg corrections to control the false discovery rate.

To compare cortical patterns of association between SNPs, the mean and standard deviation of *β* coefficients were calculated across SNPs for each cortical location. These parameters were used to standardize the coefficients by computing *z-*scores at each location. Cortical maps of standardized *β* coefficients were generated for selected SNPs. These SNPs were chosen for their meaningful patterns of association with CT or GWC and for their locations within genes linked to a neurological disorder. The *p*-value for each SNP at each cortical location was calculated using a two-tailed *t*-test on the *z-*scores, with the null hypothesis that the SNP’s *β* coefficients did not differ from the mean *β* across all other SNPs for that location. This procedure created an empiric test of significance based on the values of the 662,971 SNPs tested at this location. Statistical significance was set at *p* < 0.05 with Benjamini–Hochberg corrections to control the false discovery rate. It is important to clarify that two distinct significance tests were conducted in this study: (A) one assessing the strength of the association between each SNP and each phenotype, and (B) another using *z-*scores to evaluate the deviation of each SNP from the expected (mean) normal correlation patterns with each phenotype. These analyses were conducted twice: firstly for association with CT, and secondly for association with GWC.

SNPs were ranked according to their most significant *β* coefficient. Manhattan plots displayed the *p*-values of these correlations, aligned with their respective SNP genomic positions. All rankings used Benjamini–Hochberg corrected *p*-values. For clarity, *highest-ranked* SNPs refer to the 10 SNPs featuring the most significant *β* coefficient, while *top* SNPs refer to SNPs ranked in the top 1%. Genes associated with the top SNPs were ranked based on their number of associated top SNPs in CT or GWC. To determine the typical pattern of association for each gene, the correlations across the top SNPs linked to each gene were averaged. To quantify the deviation of these patterns from the expected (mean) pattern, each averaged gene’s SNP-based map was standardized at each cortical location. This was accomplished by computing its *z-*scores using the same standardization procedure described above. SNPs were ranked a final time according to their number of significantly associated cortical locations. *Broadest-reaching* SNPs refer to the 10 SNPs with the largest number of significantly associated cortical locations. For these SNPs, *coverage* was defined as the percentage of the cortex where association was significant. All rankings were conducted twice: first for association with CT and second for association with GWC. Cortical maps of standardized *β* coefficients were generated for notable SNPs and genes from each ranking, selected for their meaningful patterns of association.

### Linkage Disequilibrium

The ten highest-ranked SNPs according to their most significant *β* coefficient from the CT and GWC regressions were analyzed for linkage disequilibrium (LD) using ${R}^{2}$ and $D'$ measurements with PLINK software (https://www.cog-genomics.org/plink/1.9) (Purcell et al., 2007). $D'$ quantifies the degree of LD relative to its maximum possible value, indicating how much two alleles are co-inherited compared to the scenario where they are independently inherited (Lewontin, 1964). ${R}^{2}$ measures the proportion of variance in one allele that can be explained by the other, providing a measure of the strength and predictability of the LD relationship between two alleles (Hill & Robertson, 1968). Computations were visually represented on a heatmap. The same procedure was implemented for the ten broadest-reaching SNPs according to their number of significantly associated cortical locations with each phenotype.

### Dimensionality Reduction

The unstandardized (*β* coefficient) cortical correlation maps of SNPs significantly associated with CT at over 1% of cortical locations (169 SNPs) were projected into a lower-dimensional space using uniform manifold approximation and projection (UMAP) (McInnes et al., 2020). UMAP was computed using the following parameters: 15 neighbors, 2 components, a minimum distance of 0.1, and a Euclidean metric. Following UMAP embedding, we clustered SNPs using a graph-based approach. An adjacency matrix was constructed, connecting SNPs within a predefined Euclidean radius in the UMAP space. Clusters were identified using connected component analysis, which assigned SNPs to distinct groups based on their spatial relationships. The clustering radius was chosen based on the spatial organization of SNPs to ensure natural groupings without excessive fragmentation or merging. Visual inspection confirmed that UMAP effectively grouped SNPs with similar cortical association patterns, and the chosen radius successfully captured these clusters without over- or under-segmentation. To ensure robustness, clustering stability was assessed across 50 UMAP runs for genetic associations with each phenotype using the Adjusted Rand Index (ARI), which ranges from −1 (complete disagreement) to 1 (perfect agreement), with 0 indicating random chance. The groupings remained highly consistent, with a mean ARI of 0.90 for CT and 0.96 for GWC (Supplementary Figs. 3 and 4). This procedure identified clusters of SNPs whose statistical associations with CT had similar cortical patterns. To determine the typical pattern of association for each cluster, the correlations across SNPs in each cluster were averaged. To measure the deviation of these patterns from the expected mean, we standardized each cluster’s averaged SNP map at each cortical location. This was done by computing *z-*scores using the standardization procedure described earlier. These *z-*scores were plotted on cortical maps for each group. We then conducted a literature review on the genes most frequently mapped to each cluster’s SNPs to identify primary functional themes. The same procedure was implemented for SNPs significantly associated with GWC at over 1% of cortical locations (124 SNPs).

### Karyotype Plot

Karyotype plots displayed the chromosomal locations of the top 0.1% of SNPs ranked according to their most significant *β* coefficient. Each band represented one SNP. The band color indicated the Benjamini–Hochberg corrected *p*-value of the SNP’s most significant *β* coefficient. We also displayed chromosomal locations of (A) genes linked to the top 10 highest-ranked SNPs according to their most significant *β* coefficient, (B) genes linked to the top 10 broadest-reaching SNPs according to their number of significantly associated cortical locations, (C) genes most frequently linked to the top 1% of SNPs ranked according to their most significant *β* coefficient, and (D) genes most frequently linked to each UMAP cluster. Karyotype plots were produced for associations with CT and then for associations with GWC.

## Results

### Genetic Determinants of CT and GWC

**Fig. 1:**
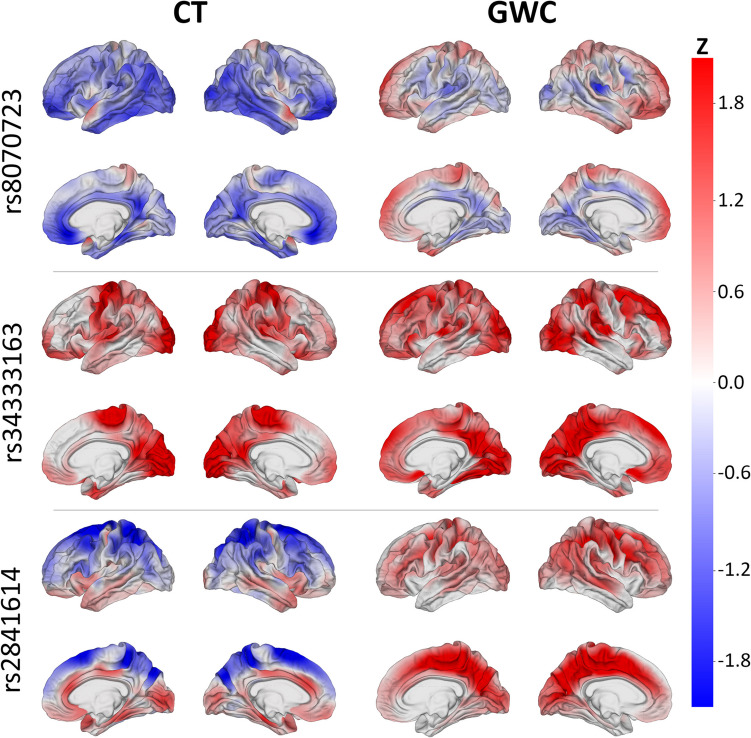
*z-*scores of SNPs’ correlations between SNPs’ reference allele numbers and two MRI-derived phenotypes (left: CT; right: GWC). Red: correlation of SNP with phenotype; blue: anticorrelation. SNPs whose maps are shown include rs8070723 (MAPT, top), rs34333163 (SLC39A8, middle), and rs2841614 (KCNK2, bottom). SNPs were chosen for their meaningful patterns of association with CT or GWC and location in a gene linked to a neurological disorder

Spatial patterns of SNP association with (A) CT and (B) GWC are displayed on cortical overlays using *z-*scores (Fig. 1). SNPs chosen for display were selected for their meaningful patterns of association with CT or GWC and for their locations within genes linked to a neurological disorder. All descriptions of spatial patterns of SNP association on the cerebral cortex are provided in comparison to the expected association for the average SNP. A significant correlation for this comparison is defined as having Benjamini–Hochberg corrected *p* < 0.05. A SNP (rs8070723) within the microtubule associated protein tau (MAPT) gene (Sollis et al., 2022) is significantly correlated with CT in the superior regions of both temporal poles and significantly anticorrelated with CT in the anterior and posterior cingulate cortices, orbitofrontal gyri, and occipital regions of both hemispheres. This SNP features significant anticorrelation with GWC in the right posterior insular cortex (Fig. 1).

The solute carrier family 39 member 8 (SLC39A8) gene contains a SNP (rs34333163) (Sollis et al., 2022) that exhibits significant correlation with CT in both precentral gyri, occipital poles, paracentral lobules, cunei, and posterior cingulate cortices (Fig. 1). This SNP is significantly correlated with GWC in the superior frontal gyri, posterior cingulate cortices, cunei, precunei, and posterior insular cortices of both hemispheres; right superior parietal lobule; and posterior region of the right superior temporal sulcus.

A SNP (rs2841614) in the potassium two pore domain channel subfamily K member 2 (KCNK2) gene (Church et al., 2011) features significant anticorrelation with CT in the superior frontal gyri, superior parietal lobules, and precunei in both hemispheres and slight correlation with CT in the limbic regions (Fig. 1). This SNP exhibits significant bilateral correlation with GWC in the superior frontal gyri, paracentral lobules, precunei, cunei, cingulate cortices, and sulci of the frontal and parietal lobes.

### GWAS

**Fig. 2:**
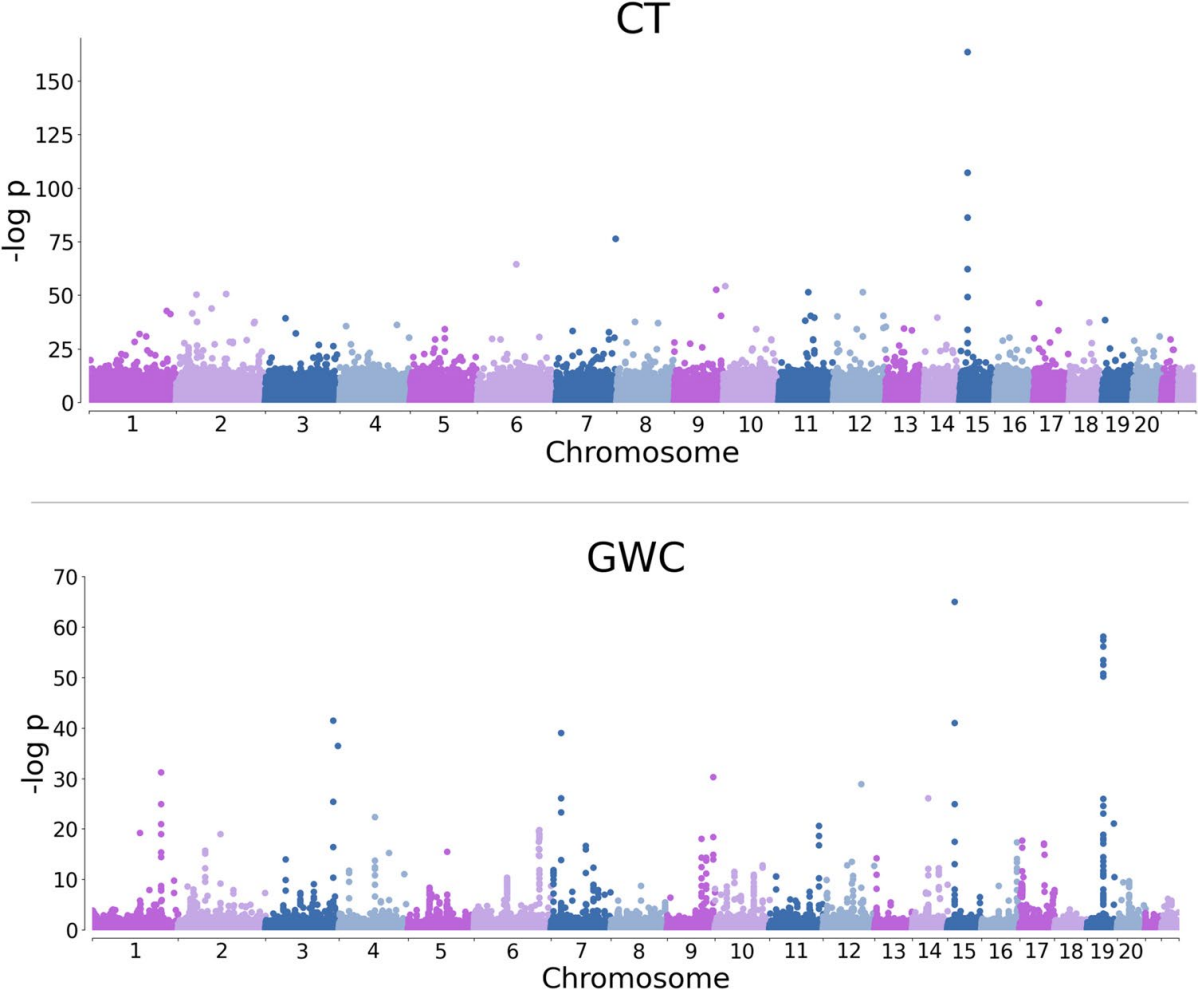
Manhattan plots for CT (top) and GWC (bottom)

Our analyses identify 169 SNPs featuring significant (*p* < 0.05) genome-wide associations with CT in at least 1% of the cortical locations examined. Additionally, 124 SNPs were significantly associated with GWC in at least 1% of cortical locations. Manhattan plots specify the statistical significance of each SNP’s most significant *β* coefficient for association with (A) CT and (B) GWC (Fig. 2, Supplementary Information 1, Supplementary Information 2). SNPs most strongly associated with CT (rs1080066, rs1837636, rs8032326, rs59203590, rs56342240, and rs72876937) are in Chromosomes 6, 7, and 15, while SNPs most strongly associated with GWC (rs1080066, rs1837636, rs10421769, rs7259333, rs10416265, rs2287679, rs10411529, rs12461219, and rs8106293) are in Chromosomes 15 and 19.

### Genetic Variants Most Strongly Associated with CT and GWC

**Fig. 3:**
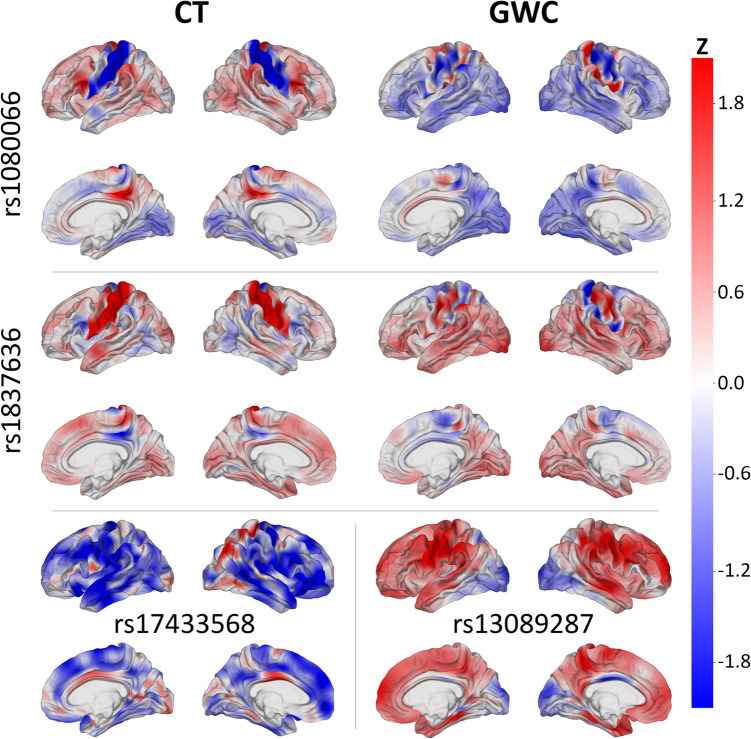
*z-*scores of SNPs’ correlations between SNPs’ reference allele numbers and two MRI-derived phenotypes (left: CT; right: GWC). Red: correlation of SNP with phenotype; blue: anticorrelation. SNPs whose maps are shown include rs1080066 (THSB1 and LINC02915, top), rs1837636 (THSB1 and LINC02915, center), rs17433568 (PTPA, bottom left), and 13089287 (OSTN, bottom right). These SNPs are among the 10 variants featuring the most significant *β* coefficient for associations with CT or GWC, respectively

Cortical maps display spatial patterns of SNP correlations with each phenotype for selected highest-ranked SNPs using *z-*scores (Fig. 3). Among the 10 highest-ranked SNPs for CT, four (rs1080066, rs1837636, rs8032326, and rs59203590) are mapped to both the thrombospondin 1 (THSB1) and long intergenic non-protein coding (LNC) RNA 2915 (LINC02915) gene (Table 1) (Sollis et al., 2022). These SNPs exhibit significant bilateral association with CT in the postcentral gyri and inferior regions of the precentral gyri. Three of these SNPs (rs1080066, rs8032326, and rs59203590) are anticorrelated in these regions, while one (rs1837636) is positively correlated (Fig. 3). The rs1080066 SNP features the most significant *β* coefficient for association with CT.

**Table 1:** Ten highest-ranked SNPs according to their most significant *β* coefficient (-log *p ≥ *51.5) for association with CT

Gene(s)	SNP	Genomic Position	β	-log p	Coverage (%)	Function
THBS1; LINC02915	rs1080066	chr15:39342021	−0.1494	163.5	7.96	modulates dendritic cell activation and cytokine release to resolve neuroinflammation; non-protein coding
	rs1837636	chr15:39369578	0.1451	107.3	5.87	
	rs8032326	chr15:39327255	−0.1178	86.3	5.37	
	rs59203590	chr15:39303639	−0.0920	62.4	4.23	
RELN	rs56342240	chr7:103594381	−0.0001	76.3	0.04	regulates neuronal migration
EYS	rs72876937	chr6:64643203	−0.0010	64.5	0.94	epidermal growth factor-like domains
Lnc-PFKP-11	rs34672417	chr10:3315471	−0.0030	54.2	0.86	non-protein coding
PTPA	rs17433568	chr9:129156623	−0.0012	52.6	0.08	accelerates protein folding
Lnc-PAWR-1	rs79213822	chr12:79484796	−0.0006	51.5	0.04	non-protein coding
TENM4	rs141706152	chr11:78738523	−0.0012	51.5	0.08	modulates connecting neurons

For CT, the reelin (RELN), eyes shut homolog (EYS), phosphotyrosyl phosphatase activator (PTPA), and teneurin transmembrane protein 4 (TENM4) gene are each mapped to one of the six other highest-ranked SNPs. The rs56342240 SNP, located in the RELN gene (Church et al., 2011), is significantly correlated with CT in the anterior cingulate cortices of both hemispheres and significantly anticorrelated with CT in both superior temporal gyri. The rs72876937 SNP is within the EYS gene (Church et al., 2011) and exhibits significant bilateral anticorrelation with CT in the medial occipitotemporal gyri and cunei. The rs141706152 SNP, located in the TENM4 gene (Church et al., 2011), features significant anticorrelation with CT in the left parahippocampal gyrus, left superior temporal sulcus, and both central sulci. The rs17433568 SNP is in the PTPA gene (Church et al., 2011) and significantly correlated with CT in both midcingulate cortices and the parietal regions of the right hemisphere (Fig. 3). This SNP also exhibits significant anticorrelation with CT in the superior and middle frontal gyri, supramarginal gyri, and temporal regions of both hemispheres.

Along with the four SNPs mapped to the LINC02915 gene, one of the highest-ranked SNPs (rs79213822) for CT belongs to the LNC RNA gene Lnc-PAWR-1 (Church et al., 2011). Another one of the highest-ranked SNPs for CT (rs34672417) has not yet been mapped to a gene but is in close proximity (3,324 bp) to Lnc-PFKP-11 (Church et al., 2011). Compared to the average SNP, the rs79213822 SNP is significantly anticorrelated with CT in the right superior temporal sulcus, left inferior temporal gyrus, and both superior frontal gyri. The rs34672417 SNP exhibits significant correlation with CT in the superior and middle frontal gyri of both hemispheres and angular gyrus of the right hemisphere.

Among the 10 highest-ranked SNPs for GWC, two (rs1080066 and rs1837636) are mapped to the THSB1 and LINC02915 genes (Table 2) (Sollis et al., 2022). These SNPs exhibit significant bilateral association with GWC in the precentral and postcentral gyri and the central sulci. The rs1080066 SNP is anticorrelated in these regions, while the rs1837636 is positively correlated (Fig. 3). The rs1080066 SNP features the most significant *β* coefficient for association with GWC.

**Table 2:** Ten highest-ranked SNPs according to their most significant *β* coefficient (-log *p ≥ *41.0) for association with GWC

Gene(s)	SNP	Genomic Position	β	-log p	Coverage (%)	Function
THBS1; LINC02915	rs1080066	chr15:39342021	−0.0050	65.1	5.27	modulates dendritic cell activation and cytokine release to resolve neuroinflammation; non-protein coding
	rs1837636	chr15:39369578	0.0060	41.0	2.97	
GPATCH1	rs10421769	chr19:33114406	0.0073	58.2	0.25	involved in mRNA splicing
	rs7259333	chr19:33088222	0.0078	57.5	0.35	
	rs10416265	chr19:33114394	0.0076	53.5	0.27	
	rs2287679	chr19:33109858	0.0076	52.5	0.31	
	rs10411529	chr19:33125350	0.0073	50.8	0.33	
RHPN2	rs12461219	chr19:33059727	0.0078	56.2	0.29	regulates stress fiber formation via the Rho pathway
	rs8106293	chr19:33045470	0.0070	50.2	0.20	
OSTN	rs13089287	chr3:190936635	0.0147	41.5	3.12	negative regulation of dendrite extension

Of the eight remaining highest-ranked SNPs for GWC, five (rs10421769, rs7259333, rs10416265, rs2287679, and rs10411529) are in the G-patch domain containing 1 (GPATCH1) gene (Church et al., 2011; Sollis et al., 2022). These SNPs are significantly correlated with GWC in the left precentral gyrus, right superior frontal gyrus, and inferior segment of the circular sulcus of the right insula. The rhophilin GTPase binding 2 (RHPN2) gene contains two of the highest-ranked SNPs (rs12461219 and rs8106293) for GWC (Church et al., 2011), which exhibit significant correlation in the same cortical regions. The final highest-ranked SNP (rs13089287) is located in the osteocrin (OSTN) gene (Sollis et al., 2022) and features significant bilateral correlation with GWC in the precentral and postcentral gyri and the central sulci (Fig. 3). This SNP is also significantly anticorrelated with GWC in the occipital poles.

### Genes Most Frequently Mapped to Variants in the Top 1% of CT and GWC Associations

**Fig. 4:**
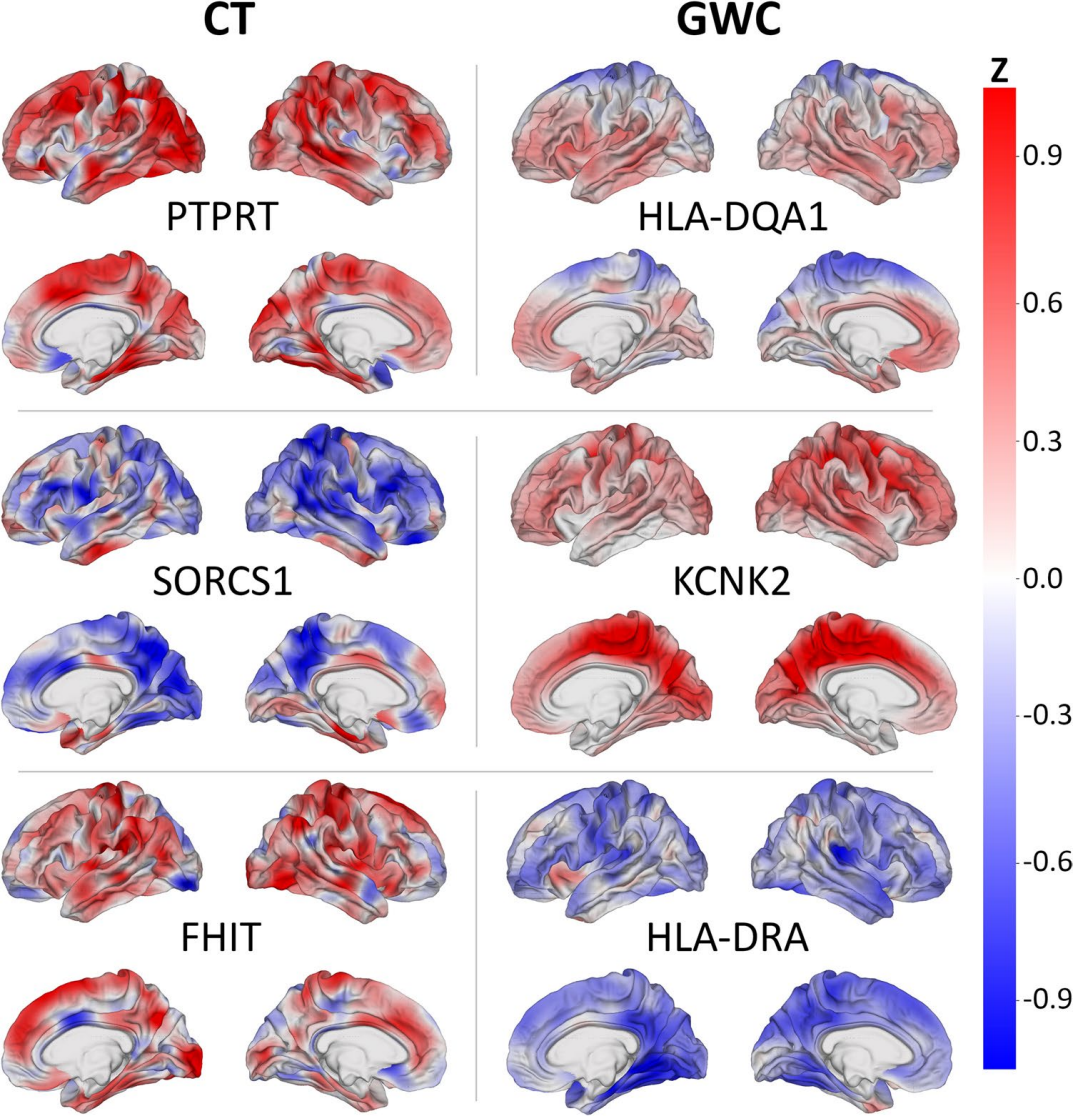
Average association between SNPs and two MRI-derived phenotypes (left: CT; right: GWC) for six genes. These genes are among the top 10 genes most frequently linked to the top 1% of SNPs (top-ranked SNPs) ranked according to their most significant *β* coefficient with each respective phenotype. Each cortical overlay maps the correlation between the phenotype and the top-ranked SNPs in the corresponding gene, averaged across SNPs. *z-*scores represent the standardized deviation from normality in the correlations between SNP reference allele numbers and the phenotype. Red indicates a positive correlation between SNPs and the phenotype, while blue indicates an anticorrelation. Genes whose maps are shown include PTPRT (top left), HLA-DQA1 (top right), SORCS1 (center left), KCNK2 (center right), FHIT (bottom left), and HLA-DRA (bottom right)

Genes mapped to top SNPs are ranked according to their number of associated top SNPs in (A) CT and (B) GWC. Top SNPs for CT are listed in Supplementary Information 3 and top SNPs for GWC are listed in Supplementary Information 4. Cortical maps display mean spatial patterns of SNP associations with (A) CT and (B) GWC for selected genes using *z-*scores (Fig. 4). The largest number of top SNPs (34) for CT are mapped to the craniofacial development protein 1 (CFDP1) gene (Table 3). When their cortical patterns are averaged across SNPs, these variants are correlated with CT in both superior frontal gyri and anticorrelated in the left posterior cingulate cortex and pars triangularis.

**Table 3:** Genes containing the top 1% of SNPs ranked according to their most significant *β* coefficient for association with CT

SNPs	Gene	Genomic Position	Peak β	-log p	Function
34	CFDP1	chr16:75327608–75467401	−0.00239	14.4	regulates cell adhesion and shape
15	NSF	chr17:44668035–44834830	−0.05816	14.4	involved in neurotransmitter release
13	LINC02915	chr15:39542870–39547046	−0.14941	163.5	non-protein coding
11	FSHR	chr2:49189296–49381654	0.02507	15.6	activates PI3K-AKT and ERK1/2 signaling pathways
10	PTPRT	chr20:40701396–41818546	−0.00003	10.4	regulates synaptogenesis and neuronal development
10	RBFOX1	chr16:6069025–7763342	−0.00003	21.2	regulates nucleokinesis of migrating cortical neurons
9	SORCS1	chr10:108333421–108924464	−0.00143	16.1	regulates neurexin and AMPA receptors trafficking
9	FHIT	chr3:59733003–61237126	−0.00002	13.5	purine metabolism and tumor suppression
8	DPYSL5	chr2:27070863–27173219	0.06161	25.6	regulates dendrite outgrowth in neural development
8	KAZN	chr1:14925181–15444539	0.00209	12.2	cell adhesion and cytoskeletal organization

The second largest number of top SNPs (15) for CT are located in the N-ethylmaleimide sensitive factor, vesicle fusing ATPase (NSF) gene. These SNPs feature bilateral anticorrelation with CT in the anterior cingulate cortices and circular sulci of the insular cortices. Additionally, 13 top SNPs for CT are mapped to the LINC02915 gene and exhibit bilateral anticorrelation with CT in the precentral and postcentral gyri and the central sulci. Of the remaining top SNPs for CT, 11 are within the follicle stimulating hormone receptor (FSHR) gene. These SNPs are correlated with CT in both lingual gyri.

The protein tyrosine phosphatase receptor type t (PTPRT) and RNA binding fox-1 homolog 1 (RBFOX1) genes each contain 10 top SNPs for CT. The SNPs mapped to the PTPRT gene exhibit correlation with CT in the superior frontal and superior temporal regions of both hemispheres and the occipital regions of the left hemisphere (Fig. 4). The SNPs within the RBFOX1 gene exhibit anticorrelation with CT in the occipital poles.

The sortilin related VPS10 domain containing receptor 1 (SORCS1) and fragile histidine triad diadenosine triphosphatase (FHIT) genes are each mapped to nine top SNPs for CT. The SNPs within the SORCS1 gene are correlated with CT in both inferior temporal gyri and anticorrelated with CT in both middle frontal gyri. These SNPs are also anticorrelated with CT in the postcentral gyrus, superior parietal lobule, supramarginal gyrus, and superior temporal gyrus of the right hemisphere (Fig. 4). The SNPs mapped to the FHIT gene exhibit correlation with CT in the postcentral gyri, inferior parietal lobules, lingual gyri, and occipital regions of both hemispheres and the paracentral lobule and cuneus of the right hemisphere. These SNPs are anticorrelated with CT in the left occipital pole and right midcingulate cortex (Fig. 4).

The dihydropyrimidinase like 5 (DPYSL5) and kazrin, periplakin interacting protein (KAZN) genes each contain eight top SNPs for CT. The SNPs located in the DPYSL5 gene are anticorrelated with CT in the right precentral gyrus and middle frontal gyrus. The SNPs mapped to the KAZN gene exhibit correlation with CT in the right supramarginal gyrus and anticorrelation with CT in both lingual gyri.

The largest number of top SNPs (30) for GWC are mapped to the major histocompatibility complex (HLA), class II, DQ alpha 1 (HLA-DQA1) gene (Table 4). When their cortical patterns are averaged across SNPs, these variants are correlated with GWC in frontal and temporal regions and anticorrelated with GWC in parietal regions (Fig. 4). The second largest number of top SNPs (23) for GWC are within the coiled-coil domain containing 91 (CCDC91) gene. These SNPs exhibit light bilateral correlation with GWC in the frontal regions. Additionally, 21 top SNPs for GWC are located in the HLA pseudogene HLA-L and are correlated with GWC in the right pars triangularis and anticorrelated with GWC in both superior parietal lobules. The Lysophosphatidic Acid Receptor 1 (LPAR1) gene contains 20 top SNPs for GWC. These SNPs exhibit bilateral correlation with GWC in the anterior insular cortices and bilateral anticorrelation with GWC in the parietal regions. Of the remaining top SNPs for GWC, 18 are within the HLA gene HLA-DRA and are moderately correlated with GWC in the left anterior insular cortex and anticorrelated with GWC in the lingual gyrus and posterior insular cortex of the right hemisphere (Fig. 4).

**Table 4:** Genes containing the top 1% of SNPs ranked according to their most significant *β* coefficient for association with GWC

SNPs	Gene	Genomic Position	Peak β	-log p	Function
30	HLA-DQA1	chr6:32605183–32611461	−0.0012	4.7	binds antigen peptides in APCs for T-cell recognition
23	CCDC91	chr12:28343389–28703099	0.0012	2.8	protein trafficking and localization to the Golgi complex
21	HLA-L	chr6:30227339–30234728	0.0015	1.6	pseudogene, non-protein coding
20	LPAR1	chr9:113635543–113801278	−0.0048	18.1	reorganization of the actin cytoskeleton
18	HLA-DRA	chr6:32407664–32412823	−0.0008	2.1	binds antigen peptides in APCs for T-cell recognition
17	KCNK2	chr1:215178885–215410433	0.0032	31.2	two-pore-domain background potassium channel
17	SUPT3H	chr6:44776794–45345671	−0.0012	10.2	histone H3 acetylation and deubiquitination
16	ZDHHC20P1	chr6:29675918–29676324	0.0008	2.5	pseudogene, non-protein coding
16	FERD3L	chr7:19184405–19185044	−0.0035	39	inhibits transcription activation by ASCL1/MASH1
15	LINC02915	chr15:39542870–39547046	−0.0050	65.1	non-protein coding

The KCNK2 and SPT3 homolog, complex component (SUPT3H) genes are each mapped to 17 top SNPs for GWC. The SNPs within the KCNK2 gene feature bilateral correlation with GWC in the precunei, paracentral lobules, and sulci of the frontal and parietal regions (Fig. 4). The cortical patterns of association with GWC for the SNPs mapped to the SUPT3H gene varied widely and their average was not observed to correlate with GWC.

The zinc finger DHHC-type containing 20P1 (ZDHHC20P1) pseudogene and FER3 like BHLH transcription factor (FERD3L) gene each contain 16 top SNPs for GWC. The LINC02915 gene is mapped to 15 top SNPs. The SNPs located in the ZDHHC20P1 pseudogene are correlated with GWC in the frontal regions and anticorrelated with GWC in the right superior parietal lobule. The SNPs mapped to the FERD3L exhibit anticorrelation with GWC in the right orbitofrontal gyrus. The SNPs within the LINC02915 gene feature bilateral correlation with GWC in the subcentral gyri and bilateral anticorrelation with GWC in the precentral and postcentral gyri.

### Genetic Variants Associated with CT and GWC in the Most Cortical Locations

**Fig. 5:**
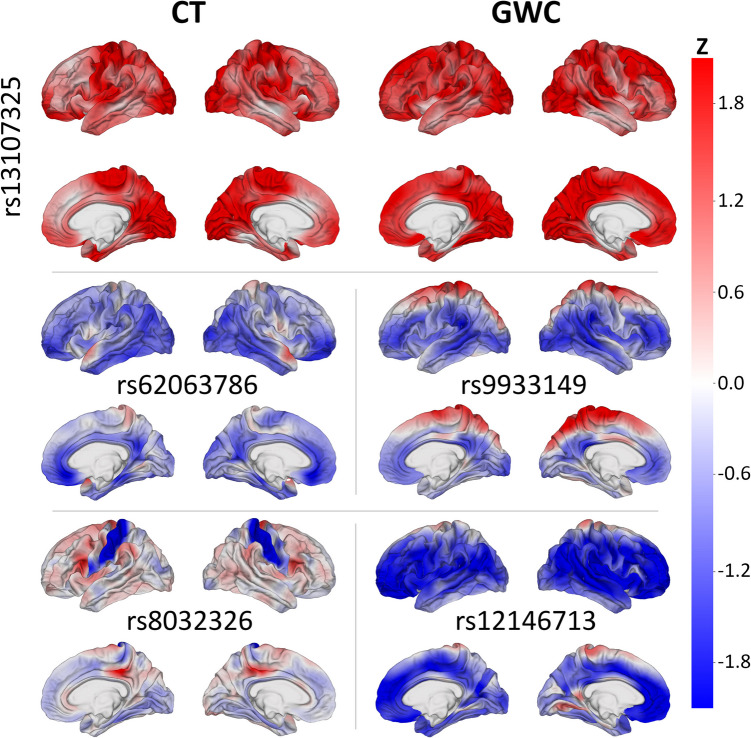
Standardized deviation from normality of SNPs’ correlations between SNPs’ reference allele numbers and two MRI-derived phenotypes (left: CT; right: GWC). Red: correlation of SNP with phenotype; blue: anticorrelation. SNPs whose maps are shown include rs13107325 (SLC39A8, top), rs62057144 (CRHR1, center left), rs9933149 (C16orf95, center right), rs8032326 (THBS1 and LINC02915, bottom left), and rs12146713 (NUAK1, bottom right). These SNPs are among the 10 variants with the largest number of significantly associated cortical locations with CT and GWC

Cortical maps display spatial patterns of SNP associations with each phenotype for selected broadest-reaching SNPs using *z-*scores (Fig. 5). Of the 10 broadest-reaching SNPs for CT, the rs13107325 SNP features the highest number of cortical locations significantly associated with CT (Table 5). This SNP is located in the SLC39A8 gene (Sollis et al., 2022) and is significantly associated with CT for over 10% of cortical locations. It exhibits significant bilateral correlation with CT in the orbitofrontal gyri, precentral and postcentral gyri, occipital regions, paracentral lobules, precunei, cunei, lingual gyri, and posterior cingulate cortices (Fig. 5).

**Table 5:** 10 broadest-reaching SNPs according to their number of cortical locations significantly associated with CT

Gene(s)	SNP	Genomic Position	Coverage (%)	Peak β	-log p	Function
SLC39A8	rs13107325	chr4:102267552	10.42	0.0567	15.1	Zn^2+^ and Mn^2+^ uptake symporter in the plasma membrane
THBS1; LINC02915	rs1080066	chr15:39342021	7.96	−0.1494	163.5	binds to ApoER2 receptor to regulate neuronal migration; non-protein coding
	rs1837636	chr15:39369578	5.87	0.1451	107.3	
	rs8032326	chr15:39327255	5.37	−0.1178	86.3	
KANSL1	rs2696671	chr17:46239522	4.82	−0.0269	15.6	regulates DNA transcription through histone H4 acetylation
CRHR1; MAPT-AS1	rs62057144	chr17:45824192	4.74	−0.0267	15.4	regulates stress-induced synthesis of serotonin; potential epigenetic regulator of MAPT expression
SPPL2C; MAPT-AS1	rs12185268	chr17:45846317	4.72	−0.0265	15.1	protease that cleaves SNARE proteins; potential epigenetic regulator of MAPT expression
MAPT	rs62063786	chr17:45983657	4.7	−0.0266	15.3	promotes microtubule assembly
	rs10445337	chr17:45990034	4.66	−0.0265	15.0	
LINC02210-CRHR1	rs2942164	chr17:45643917	4.66	−0.0267	15.4	readthrough transcript that encodes a protein identical to CRHR1

Among the nine other broadest-reaching SNPs for CT, three (rs1080066, rs1837636 and rs8032326) are mapped to the THSB1 and LINC02915 genes (Sollis et al., 2022). Their patterns of association are described above (Fig. 5). The MAPT gene contains two of the broadest-reaching SNPs for CT (rs62063786 and rs10445337) (Church et al., 2011). These SNPs are significantly anticorrelated with CT in the orbitofrontal gyri, medial and inferior temporal gyri, posterior and anterior cingulate cortices, and occipital regions of both hemispheres (Fig. 5). The SNPs also exhibit correlation with CT in the anterior regions of both superior temporal gyri but do not meet the significance threshold.

The corticotropin releasing hormone receptor 1 (CRHR1), signal peptide peptidase like 2C (SPPL2C), LINC02210-CRHR1, and KAT8 regulatory NSL complex subunit 1 (KANSL1) genes each contain one broadest-reaching SNP for CT. The SNPs located in the CHRHR1, SPPL2C, and LINC02210-CRHR1 genes (rs62057144, rs12185268, rs2942164 respectively) (Church et al., 2011) exhibit the same patterns of association as the broadest-reaching SNPs for CT located in the MAPT gene (rs62063786 and rs10445337). Two of these SNPs (rs62057144 and rs12185268) are also mapped to the MAPT-antisense RNA 1 (AS1) gene (Church et al., 2011; Sollis et al., 2022). The rs2696671 SNP, found in the KANSL1 gene (Sollis et al., 2022), is significantly anticorrelated with CT in the right anterior segment of the circular sulcus of the insula, right superior temporal sulcus, and both anterior cingulate gyri.

Among the 10 broadest-reaching SNPs for GWC, three (rs864736, rs59084003, and rs755576) belong to the KCNK2 gene (Table 6) (Church et al., 2011). These SNPs are significantly correlated with GWC throughout much of the cortex, most notably in the central sulci, superior frontal gyri, supramarginal gyri, and cingulate cortices of both hemispheres. The rs864736 SNP features the highest number of cortical locations significantly associated with GWC and is significantly associated with GWC for over 14% of cortical locations.

**Table 6:** 10 broadest-reaching SNPs according to their number of cortical locations significantly associated with GWC

Gene(s)	SNP	Genomic Position	Coverage (%)	Peak β	-log p	Function
KCNK2	rs864736	chr1:214976917	14.46	0.0032	31.2	two-pore-domain background potassium channel
	rs59084003	chr1:214980933	12.49	0.0046	24.9	
	rs755576	chr1:214961379	10.07	0.0054	21.0	
SLC39A8	rs13107325	chr4:102267552	12.69	−0.0036	22.3	Zn^2+^ and Mn^2+^ uptake symporter in the plasma membrane
PYGB	rs6115101	chr20:25248928	8.67	0.0016	9.1	regulates glycogen mobilization
NUAK1	rs12146713	chr12:106083027	8.16	−0.0237	28.9	enables p53 binding activity and protein serine/threonine kinase activity
C16orf95	rs9933149	chr16:87192600	8.1	0.0031	17.4	uncharacterized
ABHD12	rs2482923	chr20:25328345	7.92	−0.0017	9.7	mediates lysophosphatidylserine hydrolysis
	rs7267979	chr20:25317451	7.47	−0.0016	9.1	
GINS1	rs2500424	chr20:25411333	7.75	−0.0017	9.6	DNA replication and repair

Among the 6 other broadest-reaching SNPs for GWC, two (rs2482923 and rs7267979) are located in the abhydrolase domain containing 12, lysophospholipase (ABHD12) gene (Church et al., 2011). These SNPs feature significant anticorrelation with GWC in many cortical locations, primarily in the lingual gyri, insular cortices, and orbital gyri of both hemispheres.

The SLC39A8, glycogen phosphorylase B (PYGB), NUAK family kinase 1 (NUAK1), Chromosome 16 open reading frame 95 (C16orf95), and GINS complex subunit 1 (GINS1) genes each contain one broadest-reaching SNP for GWC. The rs13107325 SNP, located in the SLC39A8 gene (Sollis et al., 2022), exhibits significant correlation with GWC in many cortical locations, most notably in the occipital lobes, middle frontal gyri, posterior insular cortices, superior parietal lobules, and medial regions of both hemispheres (Fig. 5). The rs6115101 SNP is mapped to the PYGB gene (Sollis et al., 2022) and significantly correlated with GWC in the frontal regions, insular cortices, and temporal regions of both hemispheres. The rs12146713 SNP is within the NUAK1 gene (Sollis et al., 2022) and features significant bilateral anticorrelation with GWC in the frontal lobes, occipital regions, and superior and medial temporal gyri. This SNP is also correlated with GWC in the left paracentral lobule but does not meet the significance threshold for this location. The rs9933149 SNP is mapped to the C16orf95 gene (Sollis et al., 2022) and features significant bilateral correlation with GWC in the precunei and paracentral lobules. This SNP is significantly anticorrelated with GWC in the occipital regions, superior temporal sulci, supramarginal gyri, middle and inferior frontal gyri, and posterior cingulate cortices of both hemispheres. The rs2500424 SNP is in the GINS1 gene (Church et al., 2011) and significantly anticorrelated with GWC in the parahippocampal gyri, anterior insular cortices, and medial superior frontal gyri of both hemispheres and the right temporal lobe.

### Linkage Disequilibrium

**Fig. 6:**
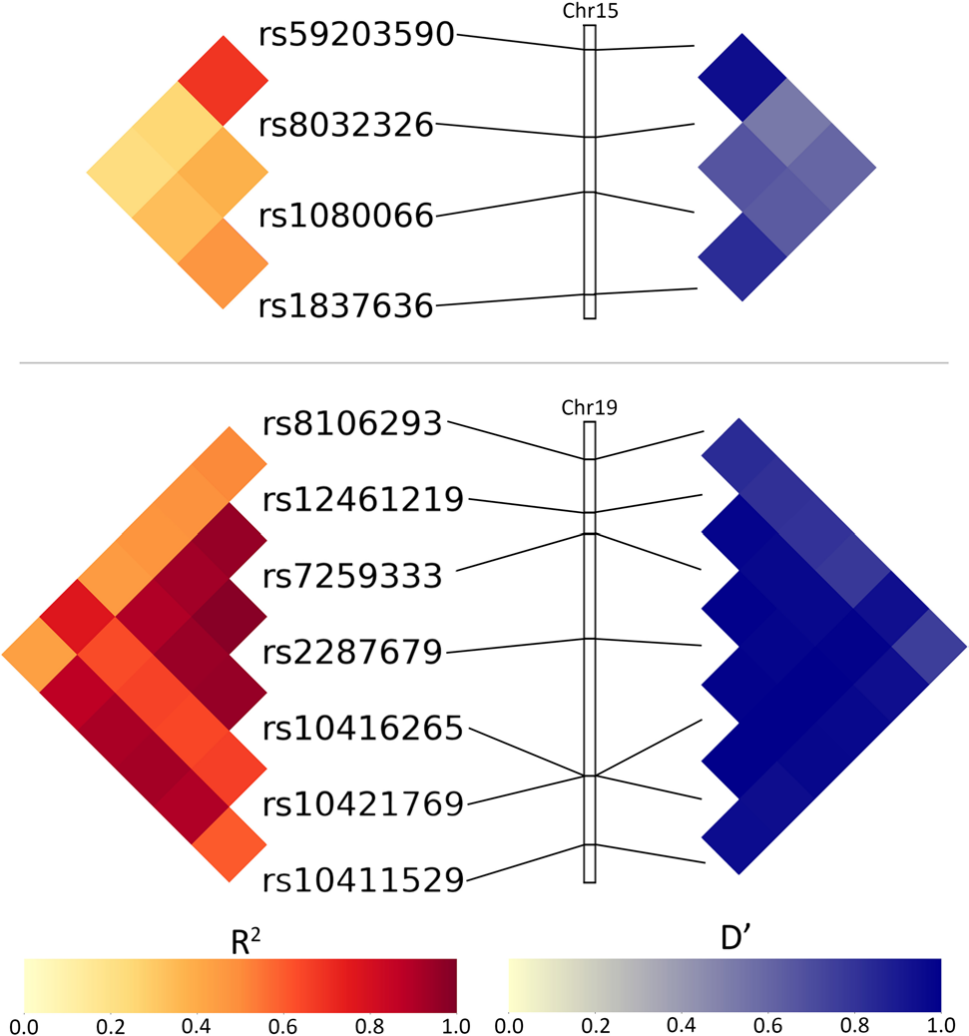
Linkage disequilibrium metrics ${R}^{2}$ (left) and $D'$ (right) for the 10 highest-ranked SNPs according to their most significant *β* coefficient with CT and GWC. All other SNPs in the top 10 for both phenotypes are located in different chromosomes. Chromosome bars represent the location of each SNP in its chromosome. The highlighted regions span from bp 39,297,044 to 39,376,171 (GRCh37) in the 15q14 cytogenic band for Chromosome 15 (top) and from bp 33,037,481 to 33,133,337 in 19q13.11 for Chromosome 19 (bottom)

**Fig. 7:**
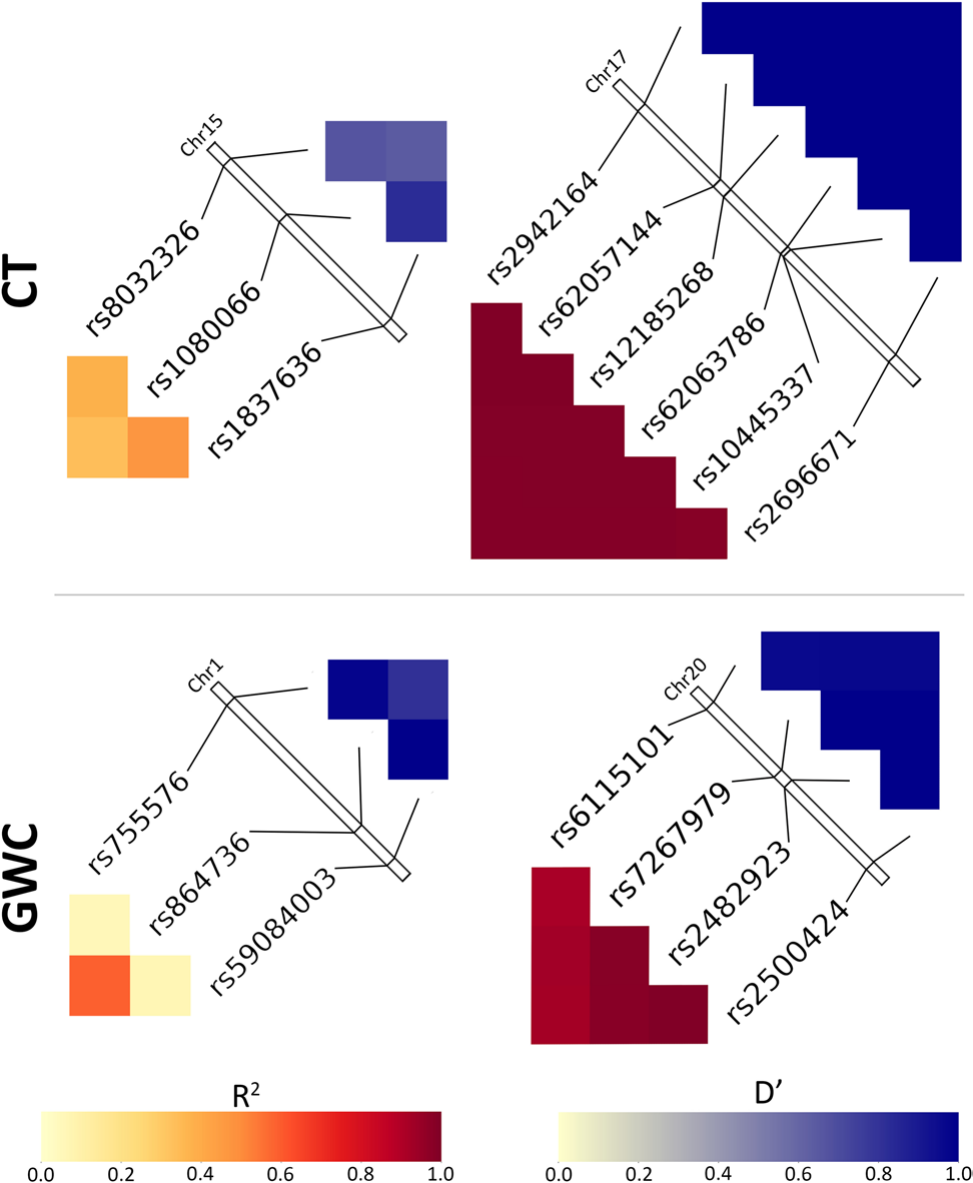
Linkage disequilibrium metrics R^2^ (left) and D′ (right) for the 10 broadest-reaching SNPs according to their number of cortical locations significantly associated with CT (top) and GWC (bottom). All other SNPs in the top 10 for both phenotypes are located in different chromosomes. Chromosome bars represent the location of each SNP in its chromosome. The highlighted regions span from bp 214,959,422 to 214,982,887 (GRCh38) in the 1q49 cytogenic band for Chromosome 1 (bottom left), bp 39,323,021 to 39,373,809 in 15q14 for Chromosome 15 (top left), bp 45,543,817 to 46,339,622 in 17q21.31 for Chromosome 17 (top right), and bp 25,232,686 to 25,427,572 in 20p11.21 for Chromosome 20 (bottom right)

${R}^{2}$ And $D'$ metrics quantify LD between pairs of highest-ranked SNPs in the same chromosome. These values are visually represented on a heatmap (Figs. 6 and 7).

Among the 10 highest-ranked SNPs according to their most significant *β* coefficient for association with CT, four are located in Chromosome 15 and six in distinct chromosomes (Table 1). In Chromosome 15, the rs59203590 and rs8032326 SNPs are in LD according to both the ${R}^{2}$ and $D'$ metrics (Fig. 6). All other SNP combinations exhibit little-to-no LD, except for the rs1837636 and rs1080066 SNPs, which exhibit moderate ${R}^{2}$ and $D'$ values.

Of the 10 highest-ranked SNPs according to their most significant *β* coefficient for association with GWC, one is located in Chromosome 3, two in Chromosome 15, and seven in Chromosome 19 (Table 2). The two SNPs located in Chromosome 15 are the rs1837636 and rs1080066 SNPs, which exhibit moderate ${R}^{2}$ and $D'$ values, as described above (Fig. 6). In Chromosome 19, six of the seven SNPs (rs12461219, rs7259333, rs2287679, rs10416265, rs10421769, and rs10411529) are in high LD according to the D’ metric. These SNPs exhibit high ${R}^{2}$ values with each other, except for the rs10421769 SNP, which has moderate ${R}^{2}$ values with the other five SNPs. The remaining SNP (rs8106293) is in moderate LD with all other SNPs except rs10421769, with which it is in LD according to both ${R}^{2}$ and $D'$ metrics.

Among the 10 broadest-reaching SNPs according to their number of cortical locations significantly associated with CT, one is located in Chromosome 4, three in Chromosome 15, and six in Chromosome 17 (Table 5). The three SNPs located in Chromosome 15 are not in LD, except for the rs1080066 and rs1837636 SNPs (Fig. 7). These two SNPs exhibit moderate ${R}^{2}$ and $D'$ values, as described above. The six SNPs (rs2942164, rs62057144, rs12185268, rs62063786, rs10445337, and rs2696671) in Chromosome 17 are in high LD, according to both ${R}^{2}$ and $D'$ metrics.

Of the 10 broadest-reaching SNPs according to their number of cortical locations significantly associated with GWC, three are located in Chromosome 1, four in Chromosome 20, and the remaining three in distinct chromosomes (Table 6). In Chromosome 1, the rs864736 SNP is in high LD with the other two SNPs (rs755576 and rs59084003) according to the $D'$ metric but exhibits low ${R}^{2}$ values (Fig. 7). Conversely, the rs755576 and rs59084003 SNPs feature a moderate ${R}^{2}$ value with each other but are not in LD according to their low $D'$ value. All four SNPs (rs6115101, rs7267979, rs2482923, and rs2500424) in Chromosome 20 are in high LD, according to both ${R}^{2}$ and $D'$ metrics.

### Dimensionality Reduction

**Fig. 8:**
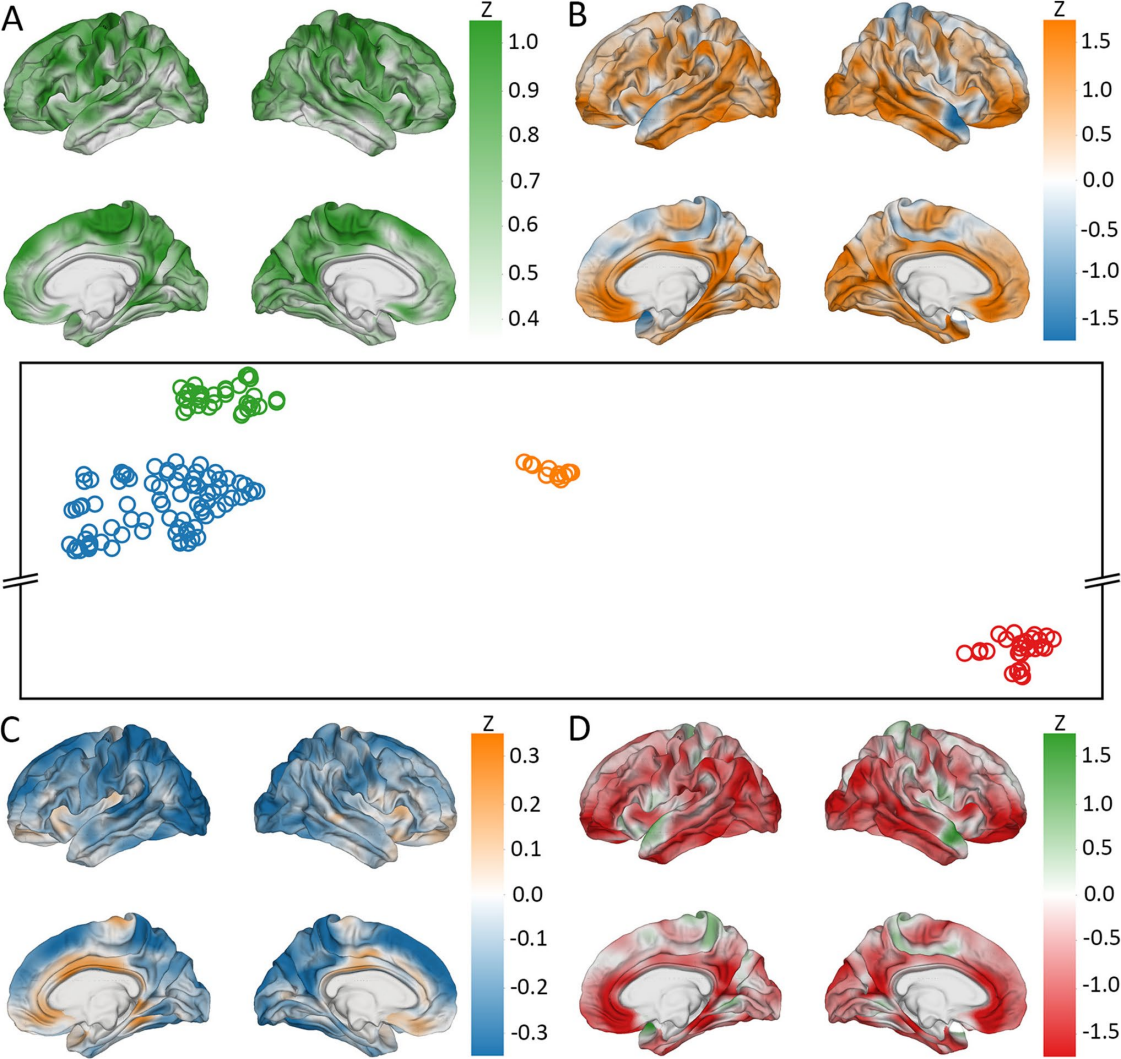
UMAP visualization of SNPs significantly associated with CT at over 1% of cortical locations. Points’ proximity within clusters indicates the extent to which SNPs’ cortical patterns of association with CT is similar across the cortex. Each cortical overlay conveys the average association between CT and the SNPs in the respective cluster. *z-*scores indicate clustered SNPs’ standardized deviation from normality of SNPs’ correlations between SNPs’ reference allele numbers and CT. Color coding (green, red, blue, and orange) links each cluster in the UMAP to its respective cortical overlay. (**A**) represents the green SNP cluster (upper left), (**B**) the orange cluster (center), (**C**) the blue cluster (lower left), and (**D**) the red cluster (bottom right)

UMAP clustered the cortical correlation maps of SNPs with CT into four groups (Fig. 8). SNPs in group A (upper left) include the broadest-reaching variant rs13107325 (SLC39A8) for CT. Genes most frequently mapped to the SNPs in group A are the SLC39A8, hyperpolarization activated cyclic nucleotide gated potassium channel 1 (HCN1), and transmembrane protein 63B (TMEM63B) genes (Table 7). When their cortical patterns are averaged across SNPs, the variants in Group A exhibit bilateral correlation with CT in the precentral gyri, paracentral lobules, precunei, and cunei.

**Table 7:** Genes most frequently linked to SNPs in each cluster produced by the CT UMAP

Grp	Gene	Genomic Position	Function
A	SLC39A8	chr4:103172198–103266639	Zn^2+^ and Mn^2+^ uptake symporter in the plasma membrane
	HCN1	chr5:45255050–45696482	hyperpolarization-activated cation channel permeable to sodium and potassium ions
	TMEM63B	chr6:44094651–44123256	mechanosensitive ion channel
B	WNT3	chr17:44839872–44896058	canonical Wnt signaling pathway, tissue patterning during cortical development
	NSF	chr17:44668035–44834830	involved in neurotransmitter release
	CRHR1	chr17:43861686–43913194	G-protein coupled receptor involved in the classical stress response signaling pathway
C	GNA12	chr7:2767739–2883942	modulates cell migration during growth via the TOR signaling cascade
	EPHA3	chr3:89156771–89531284	limit cell migration during embryonic development
	DACT1	chr14:59100779–59115039	non-canonical Wnt signaling pathway, cell migration during neurogenesis
D	ARHGAP27	chr17:43471274–43510236	activates Rho GTPases
	MAPT	chr17:43971920–44105700	microtubule assembly
	SPPL2C	chr17:43922247–43924433	protease that cleaves SNARE proteins

Genes containing the SNPs in group B (upper right) include the WNT family member 3 (WNT3), NSF, and CRHR1 genes. These variants are correlated with CT in the orbitofrontal gyri, middle temporal gyri, and occipital poles of both hemispheres and the left supramarginal gyrus. They also exhibit anticorrelation with CT in the anterior regions of the right superior temporal gyrus.

SNPs in group C (lower left) include the highest-ranked variants rs1080066 rs1837636, rs8032326 and rs59203590 (THSB1, LINC02915) for CT. Genes most frequently mapped to the SNPs in group C are the G protein subunit alpha 12 (GNA12), EPH Receptor A3 (EPHA3), and disheveled binding antagonist of *β* catenin 1 (DACT1) genes. These variants feature correlation with CT in the left midcingulate cortex. They are also anticorrelated with CT in the superior frontal gyri and occipital regions of both hemispheres and the superior parietal lobule and superior temporal gyrus of the left hemisphere.

SNPs in group D (lower right) include the broadest-reaching variants rs2696671 (KANSL1), rs2942164 (LINC02210-CRHR1), rs62057144 (CRHR1, MAPT-AS1), rs12185268 (SPPL2C, MAPT-AS1), and rs62063786 and rs10445337 (MAPT) for CT. Genes containing the SNPs in group D are the rho GTPase activating protein 27 (ARHGAP27), MAPT, and signal peptide peptidase like 2C (SPPL2C). These variants are associated with CT in the same regions as group B but with the opposite spatial pattern. They are positively correlated where Group B is anticorrelated and anticorrelated where Group B is positively correlated.

**Fig. 9:**
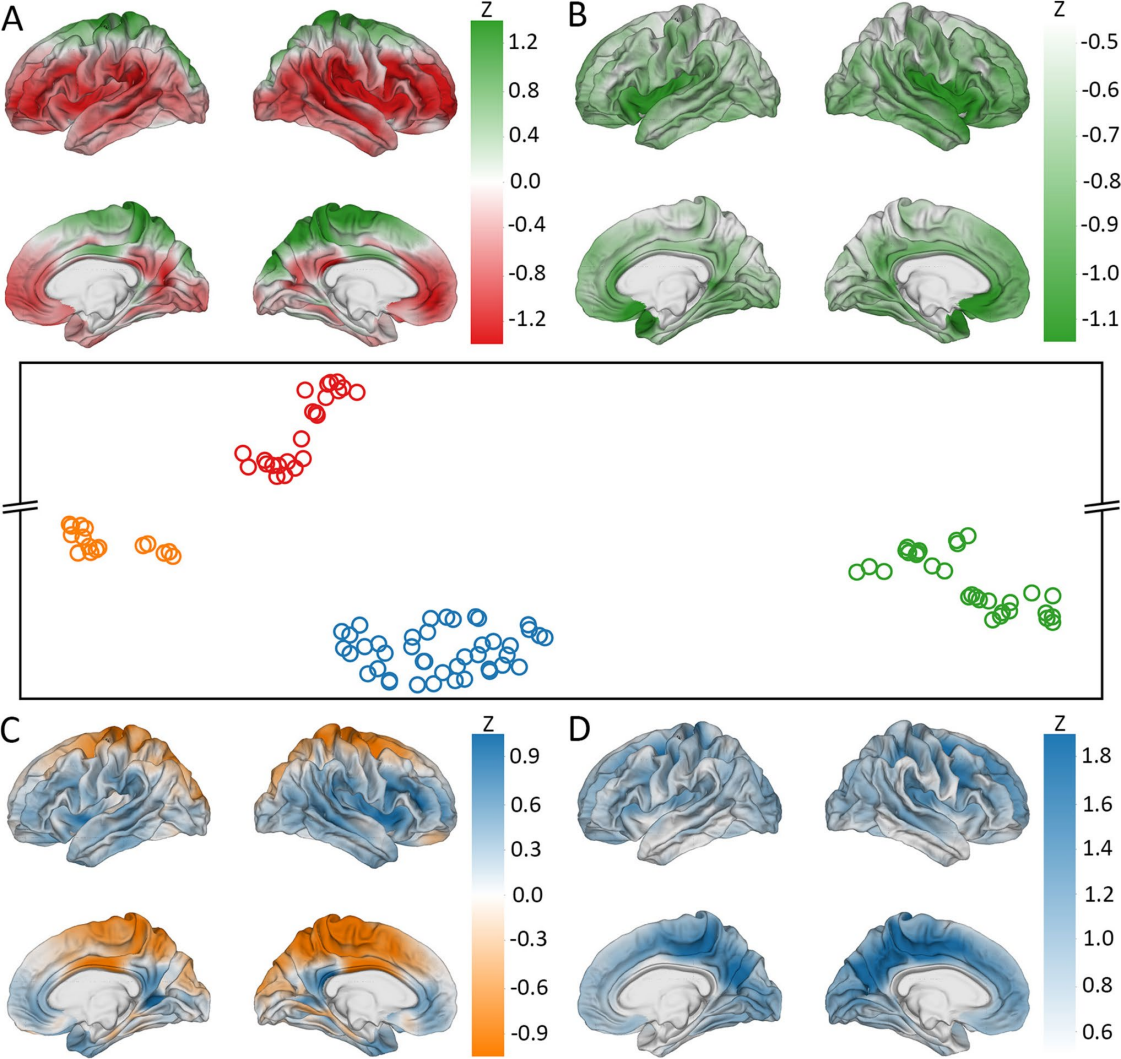
UMAP visualization of SNPs significantly associated with GWC at over 1% of cortical locations. Points’ proximity within clusters indicates the extent to which SNPs’ cortical patterns of association with GWC is similar across the cortex. Each cortical overlay conveys the average association between mean GWC and the SNPs in the respective cluster. *z-*scores indicate clustered SNPs’ standardized deviation from normality of SNPs’ correlations between SNPs’ reference allele numbers and GWC. Color coding (red, green, orange, and blue) links each cluster in the UMAP to its respective cortical overlay. (**A**) represents the red SNP cluster (upper left), (**B**) the green cluster (lower right), (**C**) the orange cluster (middle left), and (**D**) the blue cluster (bottom center)

UMAP clustered the cortical correlation maps of SNPs with GWC into four groups (Fig. 9). When their cortical patterns are averaged across variants, the SNPs in all groups feature lateralization in their correlation patterns, where SNPs are more strongly associated with GWC on the right hemisphere than the left. SNPs in group A (upper left) include the broadest-reaching variants rs12146713 (NUAK1) and rs9933149 (C16orf95) for GWC. Genes most frequently mapped to the SNPs in group A are the GNA12, LPAR1, and jagged canonical Notch ligand 1 (JAG1) genes (Table 8). These variants are correlated with GWC in the posterior superior frontal gyri, paracentral lobules, precunei, cunei, and midcingulate cortices of both hemispheres. They also exhibit bilateral anticorrelation with GWC in the sulci of the frontal regions, insular cortices, superior temporal sulci, anterior superior frontal gyri, and anterior and posterior cingulate cortices.

**Table 8:** Genes most frequently linked to SNPs in each cluster produced by the GWC UMAP

Grp	Gene	Genomic Position	Function
A	GNA12	chr7:2767739–2883942	regulates cell migration during growth through the TOR signaling cascade
	LPAR1	chr9:113635543–113801278	reorganization of the actin cytoskeleton
	JAG1	chr20:10618332–10654647	mediates Notch signaling
B	SEM1	chr7:96110938–96339158	26S proteasome responsible for degrading damaged or misfolded proteins
	NT5C2	chr10:104847774–104953029	controls purine metabolism to regulate cellular nucleotide pools
	GINS1	chr20:25388309–25429199	DNA replication and repair
C	GNA13	chr17:63005407–63052858	activates the RhoA/ROCK signaling pathway
	LPAR1	chr9:113635543–113801278	reorganization of the actin cytoskeleton
	MLLT10	chr10:21,822,685–22032559	regulates DOT1L functions and necessary for proper neuronal differentiation
D	KCNK2	chr1:215178885–215410433	two-pore-domain background potassium channel
	SLC39A8	chr4:103172198–103266639	Zn^2+^ and Mn^2+^ uptake symporter in the plasma membrane
	SLC6A20	chr3:45796941–45838028	Na^+^ and K^+^-dependent uptake of imino acids in brain

SNPs in group B (upper right) include the broadest-reaching variants rs2500424 (GINS1), rs248923 and rs7267979 (ABHD12) for GWC. Genes containing the SNPs in group B include the 26S proteasome complex subunit (SEM1), 5’-nucleotidase cytosolic II (NT5C2), and GINS1 genes. These variants are correlated with GWC in the insular cortex and medial temporal poles.

SNPs in group C (lower left) include the highest-ranked variant rs13089287 (OSTN) for GWC. Genes most frequently mapped to these SNPs include the G protein subunit alpha 13 (GNA13), LPAR1, and histone lysine methyltransferase DOT1L cofactor (MLLT10) genes. These variants are significantly associated with GWC in the same regions as group A but with the opposite spatial pattern. SNPs in Group C also exhibit weaker association with GWC in the anterior cingulate cortex when compared to Group A.

SNPs in group D (lower right) include the broadest-reaching variants rs6115101 (PYGB); rs13107325 (SLC39A8); and rs59084003, rs864736, and rs755576 (KCNK2) for GWC. Genes most frequently mapped to the SNPs in group D (lower right) include the KCNK2, SLC39A8, and solute carrier family 6 member 20 (SLC6A20) genes. These variants are correlated with GWC in the precunei and sulci of the frontal regions of both hemispheres.

### Karyotype

**Fig. 10:**
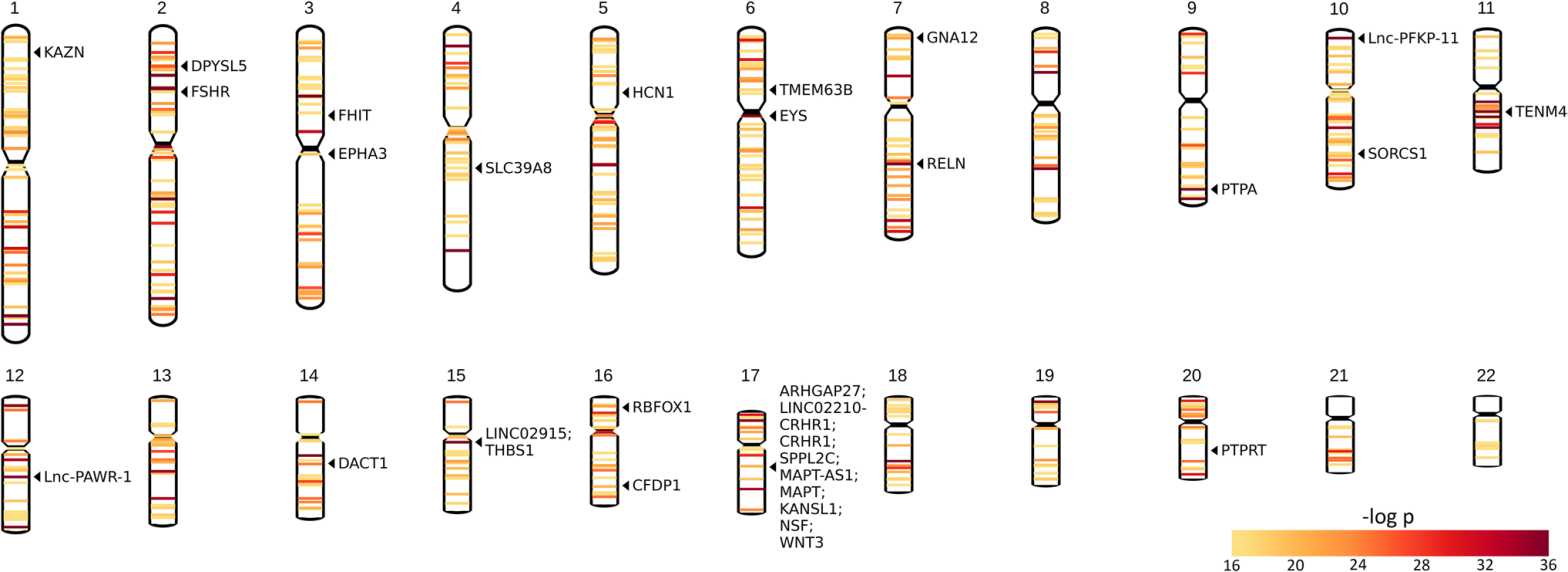
Karyotype plots displaying chromosomal location of the top 0.1% of SNPs ranked according to their most significant *β* coefficient with CT. Each band represents one SNP. The band color indicates the *p-*value of its most significant *β* coefficient. Chromosomal locations of genes linked to the top 10 highest-ranked SNPs according to their most significant *β* coefficient with CT (THBS1, LINC02915, RELN, EYS, Lnc-PFK*P-*11, PTPA, Lnc-PAWR-1, and TENM4) and number of cortical locations significantly associated with CT (SLC39A8, THBS1, LINC02915, KANSL1, CRHR1, SPPL2C, MAPT, and LINC02210-CRHR1) are displayed. Genes most frequently linked to the top 1% of SNPs ranked according to their most significant *β* coefficient with CT (CFDP1, NSF, LINC02915, FSHR, PTPRT, RBFOX1, SORCS1, FHIT, DPYSL5, and KAZN) and each cluster produced by the CT UMAP are also displayed (SLC39A8, HCN1, TMEM63B, WNT3, NSF, CRHR1, GNA12, EPHA3, DACT1, ARHGAP27, MAPT, and SPPL2C)

**Fig. 11:**
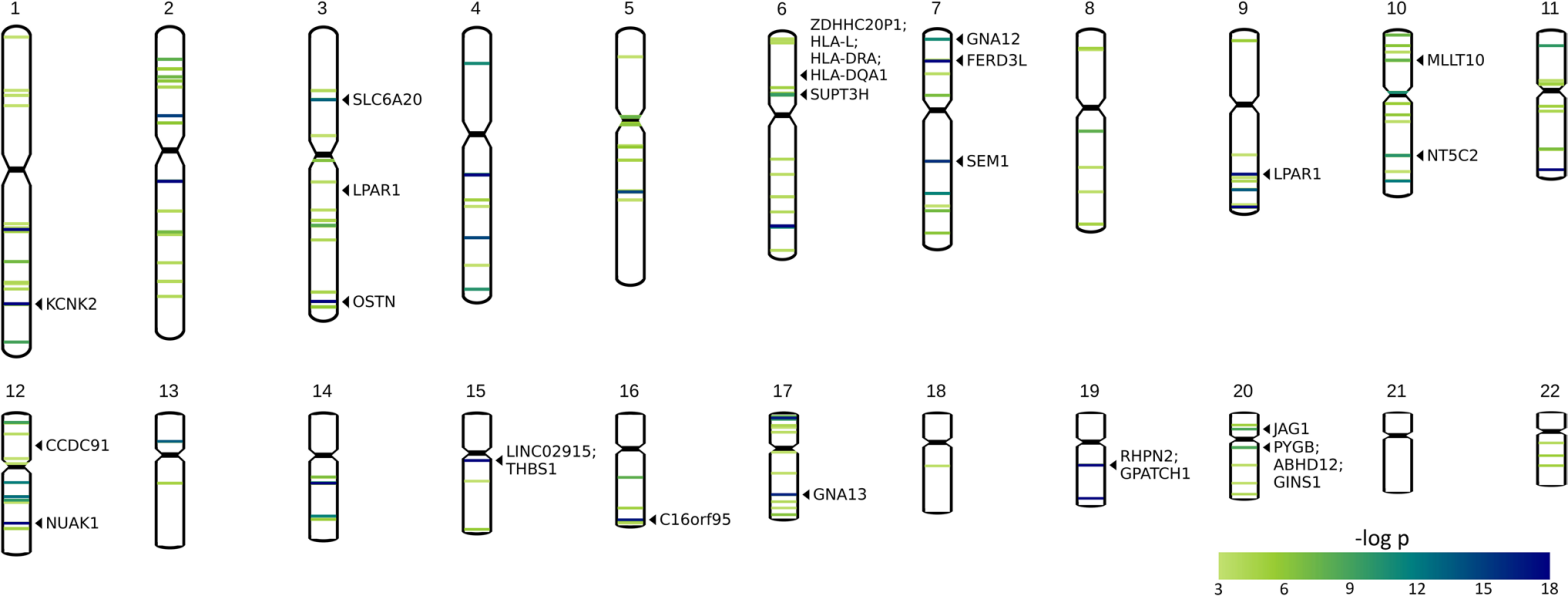
Karyotype plots displaying chromosomal location of the top 0.1% of SNPs ranked according to their most significant *β* coefficient with GWC. Each band represents one SNP. The band color indicates the *p-*value of its most significant *β* coefficient. Chromosomal locations of genes linked to the top 10 highest-ranked SNPs according to their most significant *β* coefficient with GWC (THBS1, LINC02915, GPATCH1, RHPN2, and OSTN) and number of cortical locations significantly associated with GWC (KCNK2, SLC39A8, PYGB, NUAK1, C16orf95, ABHD12, and GINS1) are displayed. Genes most frequently linked to the top 1% of SNPs ranked according to their most significant *β* coefficient with GWC (HLA-DQA1, CCDC91, HLA-L, LPAR1, HLA-DRA, KCNK2, SUPT3H, ZDHHC20P1, FERD3L, and LINC02915) and each cluster produced by the GWC UMAP are also displayed (GNA12, LPAR1, JAG1, SEM1, NT5C2, GINS1, GNA13, LPAR1, MLLT10, KCNK2, SLC39A8, and SLC6A20)

Karyotype plots display chromosomal locations of the top 0.1% of SNPs ranked according to their most significant *β* coefficient for association with (A) CT and (B) GWC (Figs. 10 and 11). Chromosomal locations of all genes mapped to significant SNPs for (A) CT and (B) GWC are also shown. SNPs in the top 0.1% for CT can be found in all 22 chromosomes. The SNPs most significantly associated with CT are located in the proximal regions of the q arms of Chromosomes 6 and 15, the interstitial regions of the q arms of Chromosome 7, 11, and 12, and the distal regions of the q arms of Chromosomes 9 and 10. Nine genes (ARHGAP27, LINC02210-CRHR1, CRHR1, SPPL2C, MAPT-AS1, MAPT, KANSL1, NSF, and WNT3) mapped to significant SNPs can be found in close proximity in Chromosome 17 in the 17q21.31 cytogenic band, spanning from base pairs (bp) 43,471,274 to 44,896,058 (Church et al., 2011).

SNPs in the top 0.1% for GWC can be found in all chromosomes except Chromosome 21. The SNPs most significantly associated with GWC are located in the proximal regions of the q arm of Chromosome 15, the interstitial regions of the q arms of Chromosomes 1, 4, 9, 12 and 19, and the distal regions of the q arm of Chromosome 3. Four genes (ZDHHC20P1, HLA-L, HLA-DRA, and HLA-DQA1) mapped to significant SNPs can be found in close proximity in Chromosome 6, spanning from bp 29,675,918 to 32,611,461 (6p22.1—6p21.32) (Church et al., 2011).

## Discussion

### Significance

We performed GWAS of CT and GWC in up to 43,002 individuals. To our knowledge, this is the first GWAS to investigate the genetic determinants of cortical *T*_*1*_*-*MRI GWC within a human population and employ UMAP to cluster cortical patterns of genetic association. Variants associated with both phenotypes are located in genes involved in neuroinflammation, neural signaling and development, stress response, and cell transport. Variants linked to CT are further mapped to genes responsible for cell migration, cytoskeleton assembly, and DNA transcription, while those associated with GWC are located in genes involved in cellular homeostasis, tissue repair, and mRNA splicing. Many of these variants and their mapped genes have been implicated in neurodegeneration by previous research. Our findings provide insight into genetic determinants of GWC and CT, highlighting pathways associated with neuroanatomy and neurodegenerative disease pathology. These insights could lay the foundation for developing gene therapies and treatments targeting neurodegenerative disease in the future.

### GWAS

We identified 42 SNPs exhibiting significant genome-wide associations with both CT and GWC in at least 1% of the cortical locations examined. Additionally, 127 SNPs were significantly linked to CT alone in at least 1% of the cortical locations, while 82 were linked to GWC. It is beyond the scope of our study to discuss each of the 251 total SNPs identified. SNPs associated with both phenotypes are located in genes responsible for neuroinflammation and ion transport. Genes mapped to SNPs linked to CT alone participate in pathways overseeing stress response, DNA transcription, and cytoskeleton assembly. Genes containing SNPs associated with GWC govern stress fiber formation and mRNA splicing.

Among our top findings is the THBS1 gene, which is mapped to four variants (rs1080066, rs1837636, rs8032326, and rs59203590) (Sollis et al., 2022) that feature notable correlations with both CT and GWC in this study. Previous research has linked these variants to imaging-derived cortical traits. The rs1080066 SNP, which we found to exhibit the strongest correlation with both CT and GWC, has been associated with cortical surface area, total cortical area, and CT (Hofer et al., 2020; van der Meer et al., 2020). The rs1837636 SNP, also one of the highest-ranked variants for both phenotypes in this study, has been implicated in regional brain volume (Zhao et al., 2019). The rs59203590 SNP, which was ranked highly for CT, has been linked to regional brain volume and vertex-wise sulcal depth (van der Meer et al., 2021; Zhao et al., 2019). The rs8032326 SNP, also one of the highest-ranked variants for CT, has been implicated in regional cortical surface area (Makowski et al., n.d.) and CT (van der Meer et al., 2020). THSB1 is responsible for negatively modulating dendritic cell activation and cytokine release to resolve neuroinflammation (Doyen et al., 2003), a process implicated in myelin repair (Goldstein et al., 2016). Because WM intensity in *T*_*1*_*-*weighted MRI is primarily due to myelin (Koenig, 1991), THBS1 may affect GWC through this mechanism. Additionally, neuroinflammation has been associated with cortical thinning (Fleischman et al., 2010), suggesting that THBS1 may also contribute to individual differences in CT through this pathway.

Our results also indicate that ion transport may impact GWC and CT. The SLC39A8 gene contains the variant (rs13107325) (Sollis et al., 2022) that achieved genome-wide significance in the largest number of cortical locations for CT and the second-largest number of cortical locations for GWC in our analysis. SLC39A8 codes for a metal cation symporter predicted to transport iron through membranes (C.-Y. Wang et al., 2012). Iron concentration in the cortex has been linked to age-related changes in brain *T*_*1*_*-*MRI by influencing the relaxation properties of brain tissue (Ogg & Steen, 1998), indicating that SLC39A8 may contribute to variations in GWC through this mechanism. Additionally, SLC39A8 may influence CT by mediating the extracellular uptake of manganese by blood–brain barrier cells (Steimle et al., 2019). Manganese accumulation has been found to increase oxidative stress and cause neuronal damage, resulting in cortical thinning (Mogi, 2023). The KCNK2 gene, which also codes for an ion transporter (Plant et al., 2012), is mapped to 17 highest-ranked variants (Schneider et al., 2016) for GWC in this study. KCNK2 encodes a two pore domain channel that contributes to passive transmembrane potassium transport (Plant et al., 2012). While the relationship between this specific potassium channel and GWC remains unclear, previous research evidence suggests that large conductance-activated potassium channels (BK channels) can preserve myelin (Rupnik et al., 2021). Additionally, deficits in inward rectifying potassium channels have been linked to myelin vacuolization in mice (Djukic et al., 2007). Future studies could investigate whether KCNK2 influences myelination by assessing its expression in oligodendrocytes and examining myelin integrity in KCNK2 knockout models.

Previous research implicates the variants in SLC39A8 and KCNK2 in imaging-derived cortical features. The rs13107325 SNP (SLC39A8 (Sollis et al., 2022)) has been associated with CT (Hermann et al., 2021; Smith et al., 2021; van der Meer et al., 2020) and linked to MRI-derived brain morphology in a multivariate omnibus test, which incorporated CT, cortical surface area, subcortical volumes, and intracranial volume (van der Meer et al., 2020). Three (rs864736, rs59084003, rs755576) of the 17 highest-ranked KCNK2 SNPs were also among the broadest-reaching variants according to their number of cortical locations significantly associated with GWC in this study. Previous research has linked all three to brain sulcal widening (Le Guen et al., 2019) and two (rs864736 and rs59084003) to CT (Le Guen et al., 2019). Two variants (rs864736 and rs755576) have also been implicated in deep learning phenotypes derived from brain imaging (Jonsson et al., 2019; Patel et al., 2024).

In this study, seven variants strongly associated with GWC are located in the RHPN2 (rs12461219 and rs8106293) and GPATCH1 genes (rs10421769, rs7259333, rs10416265, rs2287679, and rs10411529) (Schneider et al., 2016; Sollis et al., 2022). We found these SNPs to be in LD and to feature nearly identical patterns of association with GWC across the cortex. RHPN2 regulates stress fiber formation via the Rho pathway (Peck et al., 2002). Inactivation of this pathway has been shown to promote WM regrowth in the central nervous system, suggesting that RHPN2 may influence GWC through this mechanism (Lehmann et al., 1999). GPATCH1 is predicted to be involved in mRNA splicing (Bult & Sternberg, 2023), a process that affects various protein functions, including the production of myelin basic proteins (de Ferra et al., 1985; O’Connor et al., 1999). However, it remains unclear whether the mRNA splicing mediated by GPATCH1 directly affects myelin (and by extension, GWC), warranting further investigation. Future studies could explore this potential link by examining GPATCH1 expression in oligodendrocytes and assessing how its knockdown or overexpression impacts myelin-related gene regulation.

Variants mapped to RHPN2 and GPATCH1 have been linked to other cortical attributes in previous studies. Both genes were associated with (A) a SNP implicated in regional brain volume (Zhao et al., 2019), and (B) a SNP linked to MRI-derived brain morphology in a multivariate omnibus test that integrated CT, cortical surface area, subcortical volumes, and intracranial volume (van der Meer et al., 2020). Additionally, the omnibus test identified a second variant within RHPN2 associated with MRI-derived brain morphology (van der Meer et al., 2020).

We identified six variants in LD in adjacent genes that exhibited notable association with CT. Five of these SNPs (rs62063786, rs10445337, rs12185268, rs62057144, and rs2942164) featured nearly identical cortical patterns of association with CT and are located in the LINC02210-CRHR1, CRHR1, SPPL2C, MAPT-AS1, and MAPT genes (Church et al., 2011; Sollis et al., 2022). Studies have shown that three of these genes (CRHR1, MAPT, and MAPT-AS1) are highly expressed in the central nervous system, particularly in the cortex (Fagerberg et al., 2014). Although the role of CRHR1 in CT remains unclear, prior research in rats suggests that CRHR1 regulates stress-induced synthesis of serotonin (Donner et al., 2016), which has been associated with increased CT (Pillai et al., 2017). LINC02210-CRHR1 is a readthrough transcript that encodes a protein identical to the product of the CRHR1 gene (O’Leary et al., 2016). SPPL2C codes for a protease that cleaves SNARE proteins (Papadopoulou et al., 2019), which are critical for vesicular trafficking and synaptic communication (Ramakrishnan et al., 2012). Disruptions in SNARE processing by SPPL2C could impair vesicle transport and neurotransmitter release, potentially reducing synaptic density (Serrano et al., 2022), a key factor in CT (Schüz & Palm, 1989). The SNPs in CRHR1 and SPPL2C are also mapped to MAPT-AS1, a potential epigenetic regulator of MAPT expression (Coupland et al., 2016). MAPT promotes microtubule assembly (Yoshida & Goedert, 2012), essential for axonal transport and neuron architecture (Holzbaur & Scherer, 2011). Because axonal transport is key for proper synaptic function (Guedes-Dias & Holzbaur, 2019), it may influence synaptic density. However, the exact mechanism—or combination of mechanisms—that directly impacts CT remains unclear, warranting further investigation. Future studies could use single-cell RNA sequencing and spatial transcriptomics to determine how these genes are expressed across different cortical layers and assess their potential roles in synaptic density and neuronal organization.

Previous studies have implicated variants in LINC02210-CRHR1, CRHR1, SPPL2C, MAPT-AS1, and MAPT in CT and other neuroanatomical traits. The rs62063786, rs10445337, and rs12185268 SNPs, which were three of the broadest-reaching SNPs for CT in this study, have been found to be in LD with a MAPT variant strongly associated with regional brain geometry (Primus et al., 2024). Other research has linked variants in CRHR1 and LINC02210-CRHR1 to vertex-wise sulcal depth (van der Meer et al., 2021), a LINC02210-CRHR1 variant to total brain volume (Zhao et al., 2019), and a MAPT-AS1 variant to increased average CT (Grasby et al., 2020).

Among the six linked variants featuring notable association with CT, the remaining SNP (rs2696671) is mapped to the KANSL1 gene (Sollis et al., 2022). KANSL1 encodes a component of an acetyltransferase complex that regulates DNA transcription through histone H4 acetylation (Cai et al., 2010). While KANSL1 affects a broad range of proteins (O’Leary et al., 2016), its specific influence on CT remains unclear. H3 acetylation has been found to increase neurogenesis and CT (Kerimoglu et al., n.d.), but the effects of H4 acetylation on CT are not well understood. Other KANSL1 variants have also been implicated in CT (Shadrin et al., 2021; Smith et al., 2021; Zhukovsky et al., 2024), as well as subcortical volume (van der Meer et al., 2020), intracranial volume (Ikram et al., 2012), and total cortical area (Smith et al., 2021) in previous research.

### Dimensionality Reduction

Four groups of SNPs were identified in each UMAP analysis. For the CT UMAP, we found three primary functional themes: neural signaling, ion transport, and cell migration, with two groups linked to neural signaling. When averaging their cortical correlation patterns, the SNPs in the two neural signaling groups were associated with CT in the same brain regions but exhibited opposite spatial patterns. The GWC UMAP produced four distinct functional themes: neural cell development, cellular homeostasis, tissue repair, and ion transport. The groups related to neural cell development and tissue repair were associated with GWC in the same regions but displayed inverse spatial patterns.

Among the groups identified by the CT UMAP, variants in Group A are located in the SLC39A8, HCN1, and TMEM63B genes, all of which are involved in ion transport. SLC39A8 encodes a metal cation symporter that facilitates cellular uptake of zinc and manganese (Choi et al., 2018; C.-Y. Wang et al., 2012). HCN1 encodes a hyperpolarization-activated ion channel that is permeable to sodium and potassium ions (Lee & MacKinnon, 2017). These ions—zinc, manganese, sodium, and potassium—are essential for neural signaling and overall cortical function (McKenzie et al., 2023; Momin et al., 2021; Zheng et al., 2023). TMEM63B codes for a mechanosensitive ion channel (Zheng et al., 2023), which also regulates neural signaling (Momin et al., 2021) and thus supports cortical health. Variants in HCN1 and TMEM63B have been linked to developmental and epileptic encephalopathy (DEE) (McKenzie et al., 2023; Vetro et al., 2023). Although SLC39A8 has not been directly associated with DEE, mutations in this gene can cause manganese deficiency (Park et al., 2015), which has been linked to epilepsy (Grant, 2004). DEE patients often experience declining motor function (Dulac, 2001), potentially reflected in the association between CT and SNPs in Group A in the precentral gyri (home to the primary motor cortices (Banker & Tadi, 2024)) and paracentral lobules (responsible for lower limb movement (Johns, 2014, p. 3)). However, it remains unclear whether the genetic effects on ion transport directly influence CT changes in these regions, or if these changes can fully account for the symptoms of DEE. Future research could investigate this relationship by combining functional neuroimaging with electrophysiological studies to assess how these ion transport genes affect cortical excitability and motor function.

Variants in Group B are mapped to the WNT3, NSF, and CRHR1 genes, which are involved in neural signaling and are located near each other in Chromosome 17. WNT3 is part of the canonical Wnt signaling pathway, which plays a key role in tissue patterning during cortical development (Chenn, 2008). NSF is critical for neurotransmitter release, facilitating signal transmission across chemical synapses (Tolar & Pallanck, 1998). Disruptions in this process can lead to reduced synaptic density and CT (Schüz & Palm, 1989). CRHR1 encodes a G-protein coupled receptor involved in the classical stress response signaling pathway (Herman et al., 2016). While the direct link between CRHR1 and CT is unclear, chronic stress has been associated with reduced CT (Habets et al., 2011). The reason these signaling pathways influence CT with similar patterns of association remains uncertain. Variants in WNT3, NSF, and CRHR1 have been linked to PD in previous studies (Bandres-Ciga et al., 2019; Pankratz et al., 2012; Simón-Sánchez et al., 2009). Patients with REM sleep behavior disorder—a common precursor to PD (Jellinger, 2024), exhibit cortical thinning in the orbitofrontal and occipital regions (Grimaldi et al., 2023), which are the same areas where Group B variants are associated with CT. However, it is not yet clear whether this cortical thinning is primarily driven by genetic factors, the effects of REM sleep loss, or a combination of both. Future studies could use polysomnography combined with neuroimaging to assess whether sleep disturbances mediate the relationship between these genetic variants and CT alterations.

Variants in Group C are located in the GNA12, EPHA3, and DACT1 genes, all of which regulate cell migration during development. GNA12 modulates cell migration during growth via the TOR signaling cascade (Gan et al., 2012). EPHA3 activation has been shown to limit cell migration during embryonic development (Hu et al., 2009). DACT1 regulates the non-canonical Wnt signaling pathway (Carroll et al., 2024), which is responsible for regulating cell migration during neurogenesis (Alkailani et al., 2022). Cell migration allows distinct cell types generated in different brain regions to settle in the cerebral cortex during embryogenesis (Silva et al., 2019), contributing to the radial thickening of the cortex in early development (S. Wang et al., 2022).

Variants in Group D are associated with cortical thickness (CT) in the same regions as those in Group B but exhibit an opposite spatial pattern. These SNPs are mapped to the ARHGAP27, MAPT, and SPPL2C genes, which are located near the genes containing the Group B variants in Chromosome 17. Like the genes mapped to Group B, ARHGAP27, MAPT, and SPPL2C are involved in neural signaling. ARHGAP27 activates Rho GTPases (O’Leary et al., 2016), key regulators of synaptic plasticity and density (Zhang et al., 2021). MAPT encodes the tau protein, which stabilizes cytoskeletal structures in neurons (“Selective Stabilization of Tau in Axons and Microtubule-Associated Protein 2C in Cell Bodies and Dendrites Contributes to Polarized Localization of Cytoskeletal Proteins in Mature Neurons,” 1996) and acts as a scaffolding protein in phosphorylation-based signaling pathways (Mueller et al., 2021). SPPL2C encodes a protease that cleaves SNARE proteins (Papadopoulou et al., 2019), critical components of synaptic signaling (Ramakrishnan et al., 2012), potentially linking it to reduced synaptic density. Like the Group B SNPs, these variants have been associated with PD in previous studies (Do et al., 2011; Kim et al., 2024; UK Parkinson’s Disease Consortium et al., 2011). However, the reason for the opposite spatial patterns of association between the Group D and Group B variants remains unclear. Investigating whether these variants differentially affect tau phosphorylation, synaptic function, or neuroinflammation may help explain their distinct effects on CT.

Of the groups produced by the GWC UMAP, variants in Group A are located in the GNA12, LPAR1, and JAG1 genes, all of which are involved in neural cell development. GNA12 regulates cell migration during growth through the TOR signaling cascade (Gan et al., 2012). LPAR1 is involved in the reorganization of the actin cytoskeleton, influencing cell migration, differentiation, and proliferation (Sakai et al., 2013), and is essential for normal brain development (Xiao et al., 2021). JAG1 encodes a ligand that mediates Notch signaling (Cordle et al., 2008), a pathway crucial for cell fate determination and signal integration during development (Artavanis-Tsakonas et al., 1999). These neural development processes are critical for establishing neural tissue integrity, which has been shown to influence GWC (Koenig, 1991). Additionally, variants in these genes have been linked to WM microstructure differences in previous studies (Ou et al., 2023; Zhao et al., 2021), which can alter WM intensity (Harkins et al., 2016) and thus affect GWC. These SNPs are associated with GWC in the frontal and medial parietal cortical regions, which have exhibited the most significant GWC changes with aging in prior studies (Westlye et al., 2009).

Variants in Group B are mapped to the SEM1, NT5C2, and GINS1 genes, which are involved in cellular homeostasis and maintenance. SEM1 encodes a subunit of the 26S proteasome (Sone et al., 2004), responsible for degrading damaged or misfolded proteins (Bard et al., 2018). NT5C2 controls purine metabolism to regulate cellular nucleotide pools (Jordheim, 2018). GINS1 is involved in DNA replication and repair, which is vital for maintaining genomic integrity (Cottineau et al., 2017). Protein degradation, nucleotide regulation, and genomic integrity maintenance are key to sustaining cellular homeostasis and tissue integrity (Jia & Levine, 2007; Shaid et al., 2013).

Variants in Group C are associated with GWC in the same regions as those in Group A but exhibit an opposite spatial pattern. These SNPs are located in the GNA13, LPAR1, and MLLT10 genes, all of which are involved in tissue repair and maintenance. GNA13 has been shown to promote tumor cell invasion by activating the RhoA/ROCK signaling pathway (Kelly et al., 2006a, b; Yuan et al., 2016), a critical regulator of tissue response to injury (B. K. Mueller et al., 2005). MLLT10 regulates DOT1L functions (Chen et al., 2015), which include DNA damage repair (Kari et al., 2019), and is necessary for proper neuronal differentiation (Ferrari et al., 2020). Both Groups A and C contain variants mapped to the LPAR1 gene. Like the genes mapped to the Group A variants, the genes containing the Group C variants are implicated in WM microstructure measurements (Ou et al., 2023; Zhao et al., 2021). However, the reason for the opposite spatial patterns of association between the Group A and Group C variants remains unclear. Examining gene expression differences across development and aging, or investigating potential compensatory mechanisms in myelin repair, could clarify these opposing effects on GWC.

Variants in Group D are mapped to the KCNK2, SLC39A8, and SLC6A20 genes, all of which are involved in ion and solute transport. KCNK2 encodes a two-pore domain channel that facilitates passive transmembrane potassium transport (Koenig, 1991). SLC39A8 codes for a metal cation symporter that mediates the cellular uptake of zinc and manganese (Shadrin et al., 2021; van der Meer et al., 2021). SLC6A20 is responsible for the sodium- and potassium-dependent uptake of imino acids in the brain (Takanaga et al., 2005). These genes all regulate solute transport across membranes, influencing osmolality (Lopez & Hall, 2024), which can impact WM water content, a factor linked to changes in GWC (Bansal et al., 2013). Additionally, all of these genes have been associated with other WM traits in previous studies (Sargurupremraj et al., 2020; Smith et al., 2021).

## Limitations

The genetic associations were identified in a European sample of middle-aged and older adults, providing valuable insights into cortical structure and genetic influences within this demographic. However, this focus limits the generalizability of our findings to individuals of other ancestries and younger populations. Future research should examine more diverse populations to enhance broader applicability.

Additionally, our cohort was collected from multiple UKBB imaging centers (Data Field 54), which introduced variations in MRI protocols and scanners. However, FS CT measurements have been shown to be relatively robust to such variations (Fischl & Dale, 2000). This likely extends to the GWC measurements, as they were calculated using the same GM-WM boundary estimates used to compute CT. Furthermore, *T*_*1*_-MRI intensities, which can vary between scanners and subjects, were normalized using the FS MRI-normalize function, which scaled voxel intensities to ensure a consistent mean WM intensity across all subjects (Sled et al., 1998). The UK Biobank has also implemented rigorous quality control procedures to harmonize data across sites (Alfaro-Almagro et al., 2018), and prior research using UKBB data has reported minimal site-related variability in GWAS (Dor et al., 2023). Consistent with these findings, our analysis of variance quantifying the impact of imaging center on phenotypic variability indicated negligible scanner effects. To further ensure consistency, we restricted our analyses to images acquired using Siemens Skyra 3.0 T scanners with a voxel size of 1 mm × 1 mm × 1 mm and a matrix size of 208 × 256 × 256 voxels.

Another consideration is that UMAP utilizes random initialization and stochastic optimization, meaning that its embeddings are not exactly reproducible (McInnes et al., 2020). However, our general groupings were consistently reproduced across multiple runs of UMAP. Furthermore, UMAP has been shown to produce more reproducible embeddings compared to other dimensionality reduction techniques (Becht et al., 2019), while preserving both local and global data structures for meaningful representations of complex datasets (McInnes et al., 2020). It is also more robust to noisy data than alternative methods of dimensionality reduction (Becht et al., 2019).

## Conclusions

In summary, we identified 251 SNPs associated with MRI-derived cortical traits in at least 1% of cortical locations examined. Our findings expand the understanding of the genetic basis of CT and GWC, highlighting variants associated with both phenotypes in genes linked to neuroinflammation and ion transport. Additionally, variants within genes associated with stress response, DNA transcription, and cytoskeleton assembly were found to influence CT, while those linked to stress fiber formation and mRNA splicing were associated with GWC. Dimensionality reduction of significant variant associations suggests that CT is shaped by three broad mechanisms: neural signaling, ion transport, and cell migration, whereas GWC is influenced by four themes: neural cell development, cellular homeostasis, tissue repair, and ion transport.

## Supplementary Information

Below is the link to the electronic supplementary material.

**Supplementary Fig. 1** Histogram displaying the proportion of variance in CT across subjects explained by scanner site, based on analysis of variance. Scanner site accounted for an average of only 0.17% of the variance in CT across 5,124 locations, indicating minimal scanner-related effects. (PNG 69 KB)

**Supplementary Fig. 2** Histogram displaying the proportion of variance in GWC across subjects explained by scanner site, based on analysis of variance. Scanner site accounted for an average of 0.20% of the variance in GWC across 5,124 locations, further demonstrating the limited impact of scanner variability. (PNG 75 KB)

 **Supplementary** **Fig. 3 **Heatmap of ARI for 50 independent runs of UMAP and adjacency matrix clustering method for genetic associations with CT. The heatmap shows the pairwise ARI values between different runs, with ARI values loser to 1.00 indicating greater stability and similarity in clustering results. The consistently high ARI values across all runs demonstrate the robustness and reproducibility of the clustering method applied to CT GWAS data. (PNG 81 KB)

**Supplementary Fig. 4** Heatmap of the ARI for 50 independent runs of UMAP and adjacency matrix clustering method for genetic associations with GWC. The heatmap displays the pairwise ARI values between different runs, with ARI values closer to 1.00 indicating strong agreement between clustering results. The results highlight the stability and consistency of the clustering approach when applied to GWC GWAS data. (PNG 72 KB)

**Supplementary Information 1** Genome-wide significant associations (*p* < 0.05) for CT (coverage > 1% of cerebral cortex). (CSV 12 KB)

**Supplementary Information 2** Genome-wide significant associations (*p* < 0.05) for GWC (coverage > 1% of cerebral cortex). (CSV 9 KB)

**Supplementary Information 3** Top 1% of SNPs for CT by most significant beta coefficient. (CSV 438 KB)

**Supplementary Information 4** Top 1% of SNPs for GWC by most significant beta coefficient. (CSV 432 KB)

## Data Availability

Qualified researchers may access the de-identified imaging data, genetic, and demographic information used in this study through the UKBB (http://www.ukbiobank.ac.uk). Access requires an approved application and adherence to the UKBB’s data access policies. MRI data were preprocessed, and CT and GWC values were mapped onto an average cortical atlas using FS (https://surfer.nmr.mgh.harvard.edu). LD analysis was conducted using PLINK software (https://www.cog-genomics.org/plink/1.9). Post-GWAS analyses incorporated resources from the NHGRI-EBI GWAS Catalog (https://www.ebi.ac.uk/gwas) and UMAP for dimensionality reduction (https://github.com/lmcinnes/umap). Our GWAS data is provided within the manuscript and supplementary information files.
