# Supplementary figures and images for: Genetic Insights into Brain Morphology: a Genome-Wide Association Study of Cortical Thickness and T1-Weighted MRI Gray Matter-White Matter Intensity Contrast
Source: Neuroinformatics. 2025 Apr 1;23(2):26. doi: 10.1007/s12021-025-09722-9 (PMC11961481; doi:10.1007/s12021-025-09722-9)

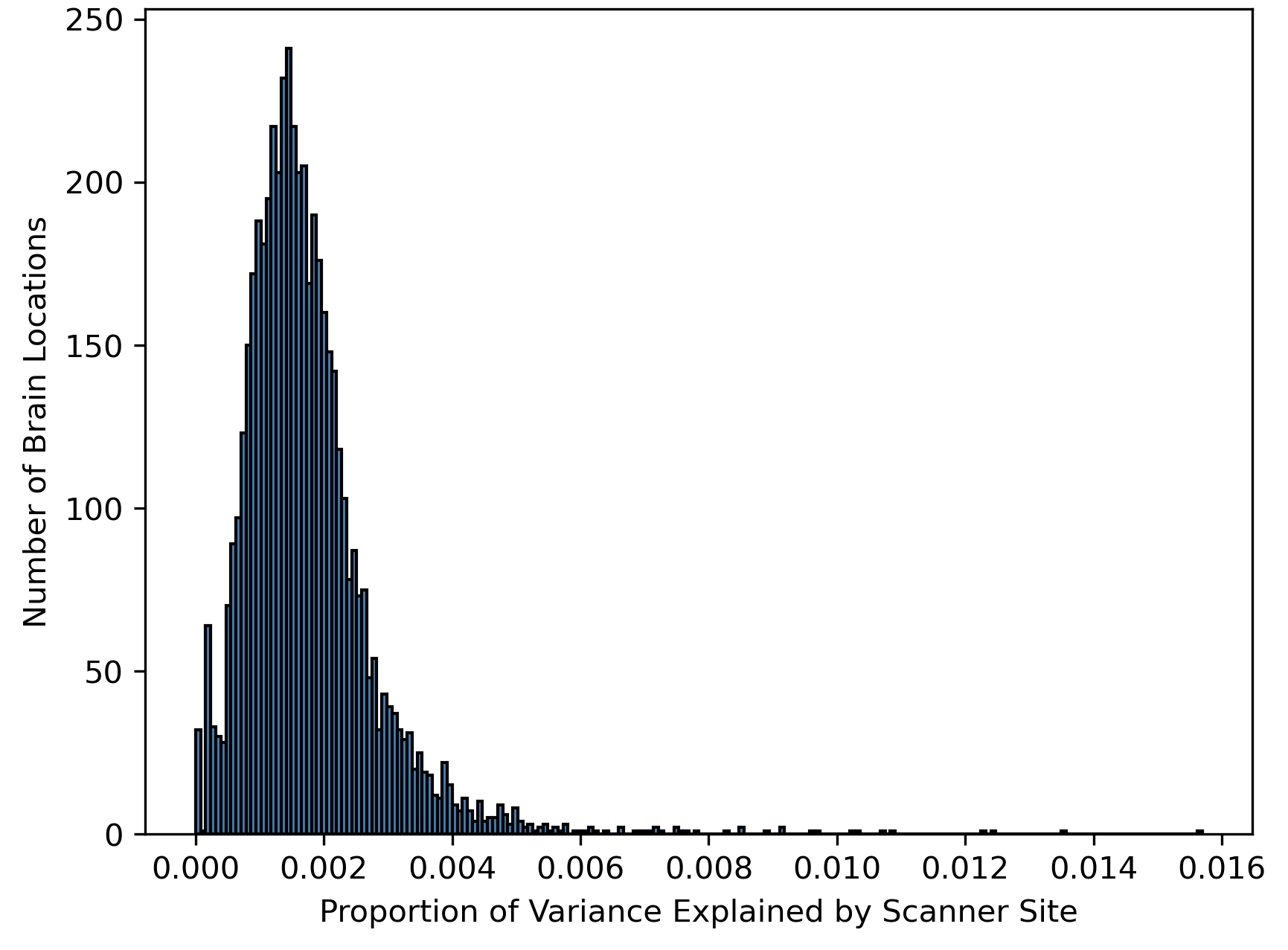

Supplement: Supplementary file 1 — Supplementary Fig. 1 Histogram displaying the proportion of variance in CT across subjects explained by scanner site, based on analysis of variance. Scanner site accounted for an average of only 0.17% of the variance in CT across 5,124 locations, indicating minimal scanner-related effects. (PNG 69 KB) [file 12021_2025_9722_MOESM1_ESM.png]

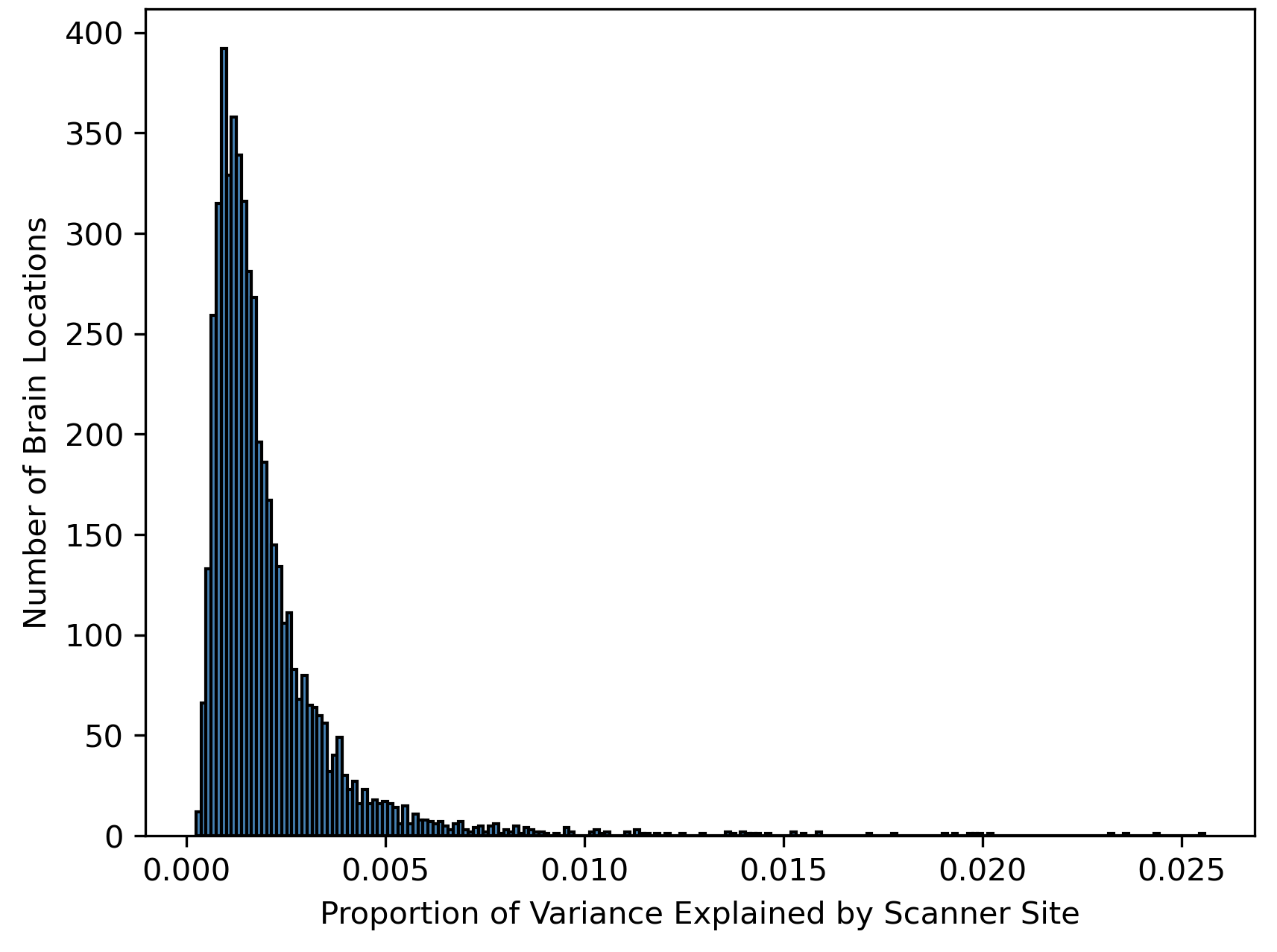

Supplement: Supplementary file 2 — Supplementary Fig. 2 Histogram displaying the proportion of variance in GWC across subjects explained by scanner site, based on analysis of variance. Scanner site accounted for an average of 0.20% of the variance in GWC across 5,124 locations, further demonstrating the limited impact of scanner variability. (PNG 75 KB) [file 12021_2025_9722_MOESM2_ESM.png]

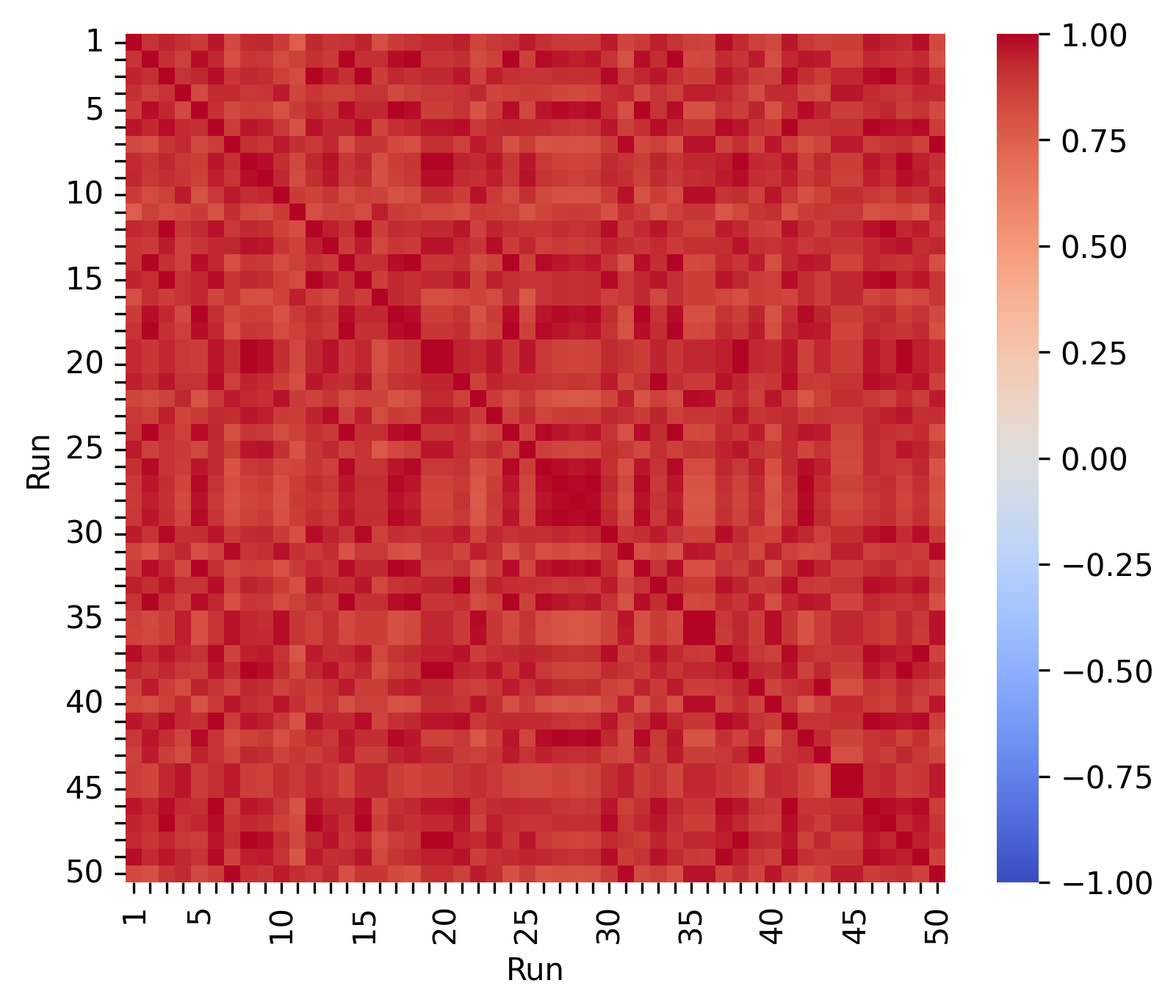

Supplement: Supplementary file 3 — Supplementary Fig. 3 Heatmap of ARI for 50 independent runs of UMAP and adjacency matrix clustering method for genetic associations with CT. The heatmap shows the pairwise ARI values between different runs, with ARI values loser to 1.00 indicating greater stability and similarity in clustering results. The consistently high ARI values across all runs demonstrate the robustness and reproducibility of the clustering method applied to CT GWAS data. (PNG 81 KB) [file 12021_2025_9722_MOESM3_ESM.png]

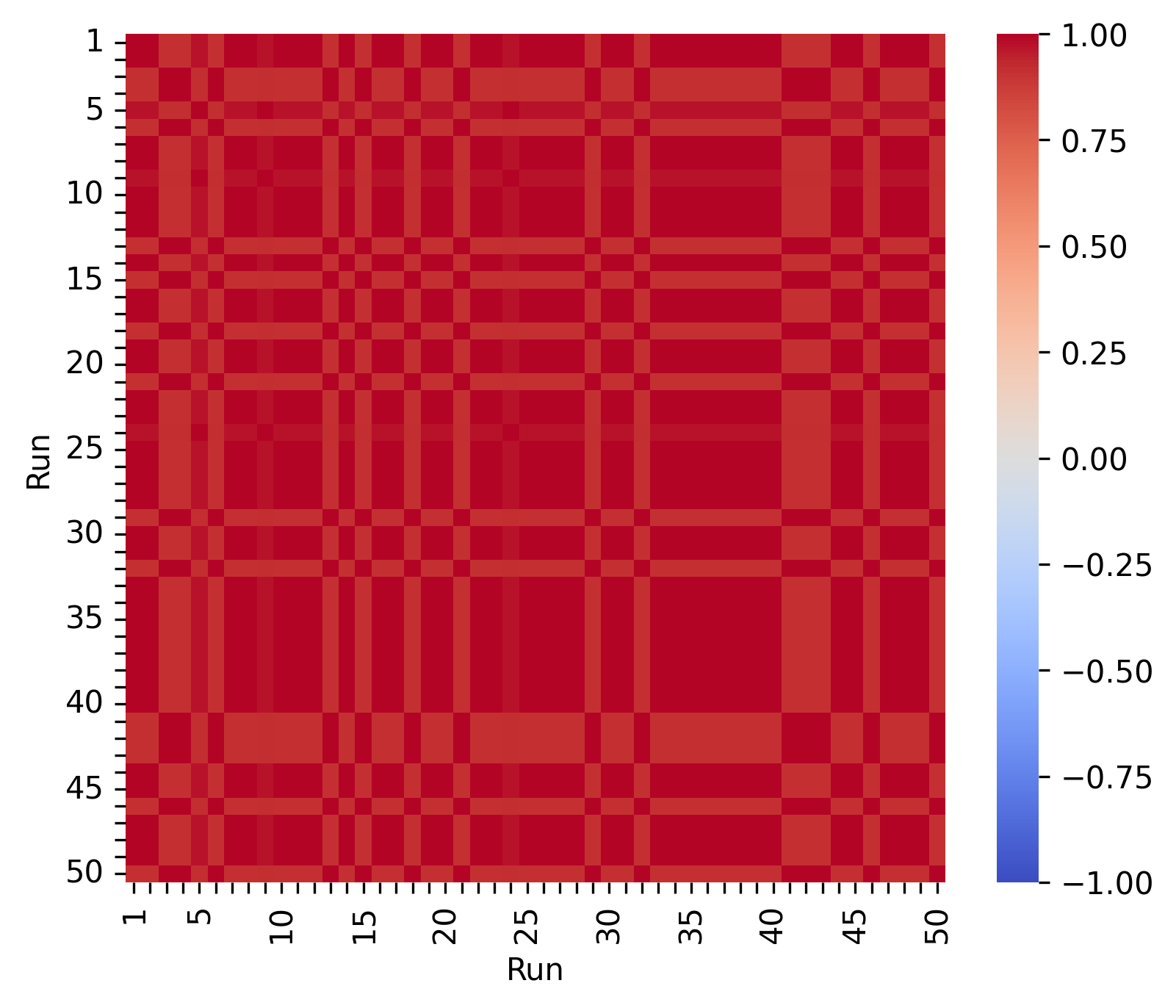

Supplement: Supplementary file 4 — Supplementary Fig. 4 Heatmap of the ARI for 50 independent runs of UMAP and adjacency matrix clustering method for genetic associations with GWC. The heatmap displays the pairwise ARI values between different runs, with ARI values closer to 1.00 indicating strong agreement between clustering results. The results highlight the stability and consistency of the clustering approach when applied to GWC GWAS data. (PNG 72 KB) [file 12021_2025_9722_MOESM4_ESM.png]
